# Unlocking the full potential of nanopore sequencing: tips, tricks, and advanced data analysis techniques

**DOI:** 10.1093/nar/gkag023

**Published:** 2026-02-02

**Authors:** Daria Meyer, Winfried Goettsch, Jannes Spangenberg, Bettina Stieber, Sebastian Krautwurst, Martin Hölzer, Christian Brandt, Jörg Linde, Christian Höner zu Siederdissen, Akash Srivastava, Milena Zarkovic, Damian Wollny, Manja Marz

**Affiliations:** RNA Bioinformatics and High-Throughput Analysis, Friedrich Schiller University, Leutragraben 1, 07743 Jena, Germany; Oncgnostics GmbH, Löbstedter Str. 41, 07749 Jena, Germany; RNA Bioinformatics and High-Throughput Analysis, Friedrich Schiller University, Leutragraben 1, 07743 Jena, Germany; Leibniz Institute on Aging - Fritz Lipmann Institute, Beutenbergstraße 11, 07745 Jena, Germany; RNA Bioinformatics and High-Throughput Analysis, Friedrich Schiller University, Leutragraben 1, 07743 Jena, Germany; Oncgnostics GmbH, Löbstedter Str. 41, 07749 Jena, Germany; RNA Bioinformatics and High-Throughput Analysis, Friedrich Schiller University, Leutragraben 1, 07743 Jena, Germany; European Virus Bioinformatics Center, Friedrich Schiller University, Leutragraben 1, 07743 Jena, Germany; Friedrich Schiller University, Institute of Microbiology, 07745 Jena, Germany; European Virus Bioinformatics Center, Friedrich Schiller University, Leutragraben 1, 07743 Jena, Germany; Genome Competence Center (MF1), Robert Koch Institute, 13353 Berlin, Germany; Institute of Infectious Diseases and Infection Control, Jena University Hospital, Friedrich Schiller University, 07747 Jena, Germany; European Virus Bioinformatics Center, Friedrich Schiller University, Leutragraben 1, 07743 Jena, Germany; Institute of Bacterial Infections and Zoonoses, Friedrich-Löffler-Institute, Naumburger Str. 96a, 07743 Jena, Germany; RNA Bioinformatics and High-Throughput Analysis, Friedrich Schiller University, Leutragraben 1, 07743 Jena, Germany; RNA Bioinformatics and High-Throughput Analysis, Friedrich Schiller University, Leutragraben 1, 07743 Jena, Germany; Leibniz Institute on Aging - Fritz Lipmann Institute, Beutenbergstraße 11, 07745 Jena, Germany; RNA Bioinformatics and High-Throughput Analysis, Friedrich Schiller University, Leutragraben 1, 07743 Jena, Germany; Leibniz Institute on Aging - Fritz Lipmann Institute, Beutenbergstraße 11, 07745 Jena, Germany; RNA Bioinformatics and High-Throughput Analysis, Friedrich Schiller University, Leutragraben 1, 07743 Jena, Germany; Leibniz Institute on Aging - Fritz Lipmann Institute, Beutenbergstraße 11, 07745 Jena, Germany; RNA Bioinformatics and High-Throughput Analysis, Friedrich Schiller University, Leutragraben 1, 07743 Jena, Germany; Leibniz Institute on Aging - Fritz Lipmann Institute, Beutenbergstraße 11, 07745 Jena, Germany; European Virus Bioinformatics Center, Friedrich Schiller University, Leutragraben 1, 07743 Jena, Germany; Max Planck Institute for Evolutionary Anthropology, Deutscher Pl. 6, 04103 Leipzig, Germany; German Center for Integrative Biodiversity Research, Puschstraße 4, 04103 Leipzig, Germany; Michael Stifel Center Jena, Friedrich Schiller University, Ernst-Abbe-Platz 2, 07743 Jena, Germany; Cluster of Excellence ‘Balance of the Microverse’, Friedrich Schiller University, Fürstengraben 1, 07743 Jena, Germany

## Abstract

Nucleic acid sequencing is the process of identifying the sequence of DNA or RNA, with DNA used for genomes and RNA for transcriptomes. Deciphering this information has the potential to greatly advance our understanding of genomic features and cellular functions. In comparison to other available sequencing methods, nanopore sequencing stands out due to its unique advantages of processing long nucleic acid strands in real time, within a small portable device, enabling the rapid analysis of samples in diverse settings. Evolving over the past decade, nanopore sequencing remains in a state of ongoing development and refinement, resulting in persistent challenges in protocols and technology. This article employs an interdisciplinary approach, evaluating experimental and computational methods to address critical gaps in our understanding in order to maximize the information gain from this advancing technology. Here, we present both overview and analysis of all aspects of nanopore sequencing by providing statistically supported insights. Thus, we aim to provide fresh perspectives on nanopore sequencing and give comprehensive guidelines for the diverse challenges that frequently impede optimal experimental outcomes.

## Graphical Abstract

**Graphical Abstract:**
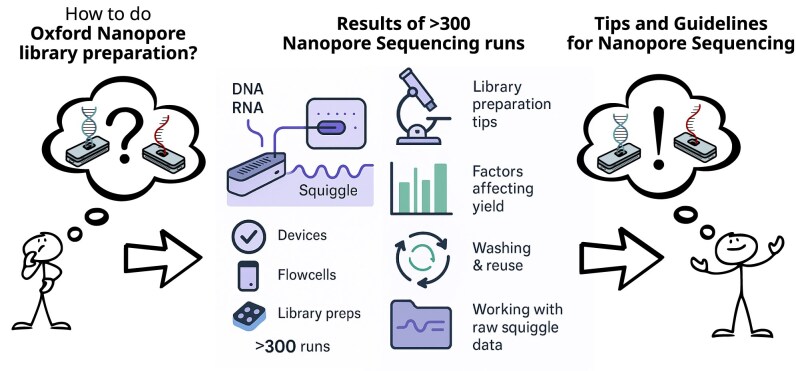

## Introduction

Nanopore sequencing is a transformative technology in genomics, offering the unique ability to sequence DNA or RNA molecules in their native form. Nanopore sequencing can generate long sequencing reads by measuring disturbances in the ion current as biological molecules such as DNA and RNA pass through a nanopore [1, 2]. During or after sequencing, the raw DNA/RNA signal can be transformed into nucleotide sequences. This capability provides invaluable insights into genetic variation and molecular modifications. Since the debut of the MinION device by Oxford Nanopore Technologies (ONT) in 2014, nanopore sequencing has experienced a surge in popularity and has become a valuable technique in genomic research. For DNA sequencing [2], it is increasingly applied in diagnostics [3–5], whole genome assembly [6], and for metagenomic assemblies [7–10], enabling the study of microbial communities and their functions. In direct RNA sequencing, it supports diverse tasks such as *de novo* transcriptome assembly, isoform expression quantification [11], and the direct detection of RNA modifications [12]. These applications highlight the technology’s versatility and its ability to address a wide range of biological questions.

One of the key advantages of nanopore sequencing is its ability to generate data rapidly, facilitated by quick library preparation and real-time data acquisition during sequencing [13]. Additionally, nanopore sequencing can directly sequence native RNA molecules without the need for reverse transcription or amplification [11, 14]. Its capacity for long-read sequencing, where fragments up to two megabases in length can be read in a single pass [15], further enhances its utility by providing comprehensive genomic and transcriptomic insights. Furthermore, due to their portability and affordability, devices such as the MinION [16] are particularly valuable for applications such as monitoring virus outbreaks, where fast and on-site sequencing is essential.

Nanopore sequencing offers many advantages but comes with technical challenges. When first introduced, the technology was limited by lower accuracy ($\sim$60%) and throughput, though these shortcomings have improved significantly over time [17]. With the introduction of ONT’s MinION device in 2014, accuracy improved to 89.5% [18], with current rates reaching 99% [19]. While in 2021 nanopore sequencing continued to exhibit a higher error rate compared to Illumina sequencing [2], in 2024 sequencing of nearly complete bacterial genomes without short-read or reference polishing became possible [20, 21]. Nonetheless, sequencing accuracy remains a key consideration for certain high-precision applications, particularly in direct RNA sequencing [22].

Another challenge lies in the customization of nanopore wet lab workflows. While numerous reviews provide detailed overviews of tools for standard nanopore sequencing applications [13, 23–25], the field’s relative novelty has given rise to a broad range of non-standard approaches aimed at maximizing sample utility. The versatility of nanopore sequencing lends itself to customization to meet specific requirements, including the use of various flow cells, library preparation kits, sequencing buffers, and an extensive set of computational analysis tools. The sheer number of options can be daunting for newcomers. Optimizing protocols, designing libraries, and selecting parameters require careful consideration. Compounding this issue is the prevalence of unverified claims in the field, as many methods lack experimental validation, making them challenging to integrate into standardized workflows. This is particularly problematic when information is available solely through the Nanopore Community (https://community.nanoporetech.com) and lacks proper citation.

This perspective work integrates insights gained from over 300 nanopore sequencing runs, which span a variety of species, sequencing devices, preparation methods, and sequencing protocols. These data-driven findings provide valuable guidance for addressing common challenges in nanopore sequencing. For instance, we show that sequencing performance is influenced more by the number of starting pores and sample type than by read length, flow cell age, or the amount of library loaded onto the flow cell. Techniques such as flow cell washing and adaptive sampling [26] have been demonstrated to enhance output and improve sequencing yield. Additionally, we offer practical advice for optimizing library preparation, particularly for obtaining long reads and adjusting loading amounts when working with small sample sizes. We also address the complexities of ONT data analysis, providing guidelines for constructing customized bioinformatics pipelines that align with specific experimental goals. Effective methods for calling DNA and RNA modifications, as well as strategies for normalizing raw signal data, are also discussed. By bridging the gap between experimental data and best practices, this review intends to equip researchers with actionable strategies for optimizing nanopore sequencing experiments.

## Background on nanopore hardware

**Figure 1.**
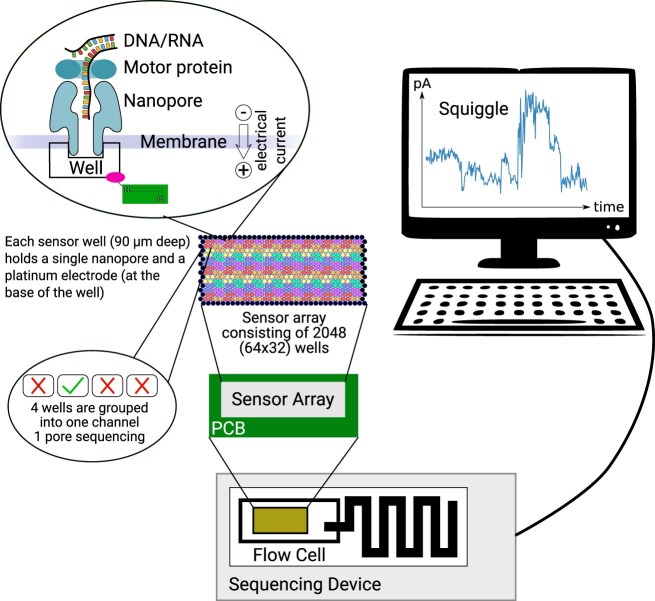
Nanopore sequencing on a MinION. A MinION flow cell contains 512 channels with 4 nanopores in each channel, resulting in 2048 nanopores available for sequencing DNA or RNA. Each sensor well (90 μm deep) holds a single nanopore and a platinum electrode (at the base of the well). The electrode is controlled via the printed circuit board and measures the ion flow as ionic current. During sequencing, a motor protein guides the DNA/RNA through the nanopore following the electric gradient. The DNA/RNA disrupts the current by partially blocking the ion flow. This can be measured in a resulting squiggle.

At the heart of nanopore sequencing is a protein channel (nanopore) through which DNA or RNA molecules can pass (Fig. 1). Electrical sensors are sensitive to electrical changes as molecules move through the nanopore [27]. Flow cells are filled with an electrolyte solution that establishes a relatively constant electrical current through the nanopore in the absence of DNA or RNA. The sensor continuously records this current as a raw electrical signal in picoamperes (pA) [28–30]. During library preparation, a motor protein is attached to the nucleotide strand, which guides the DNA or RNA molecule though the pore and unwinds it while controlling the pace at which the strand passes through, ensuring accurate sequencing [2]. As each DNA or RNA molecule passes through a nanopore, it disrupts the electrical current in a unique way based on the sequence moving through the pore. In R10 and RNA flowcells, 9 nt in the nanopore generate the signal, while in older R9 flowcells, 5 nt contributed [31, 32]. The recorded electrical signal from the sensor, frequently referred to as the “squiggle,” contains the data used in downstream processing [13].

Each flow cell contains hundreds to thousands of nanopores, which are grouped into channels of four pores. Flow cells are the standard, full-sized sequencing units, which generate tens to hundreds of gigabases (Gb) of data [33].

Different nanopore sequencing devices exist, including the MinION, GridION, P2, and the PromethION. Combined with their respective flow cell(s), ranging from low throughput and yield Flongles to high throughput and yield PromethION flow cells, they enable sequencing for a wide variety of use cases. A standard MinION flow cell has 2048 pores organized in 512 channels [2].

Flow cells differ in the design of the nanopores, which impacts their performance mainly in terms of sequencing accuracy [34]. In this work, we used R9 and R10 flow cells. R9 flowcells use single-constriction pores, which were designed to be robust, reliable, and capable of handling a wide range of sequencing tasks.

The R10 flow cells feature double-constriction pores, where the nucleotide strand passes through two constriction points instead of one. The double-constriction design enhances the resolution of basecalling and can achieve higher raw sequencing accuracy [35]. At the start of each sequencing run, an initial flow cell check is performed, providing the starting number of available pores [36, 37]. Each pore can only process a limited number of molecules before it becomes inactive. Therefore, the number of available pores decreases over time [35, 36]. Usually, even a new flow cell never has the maximum number of pores available. A warranty is provided by ONT that allows flow cells to be returned if they do not have enough available pores within 3 months of purchase (https://nanoporetech.com/document/flow-cell-check).

The pores in the flow cell are controlled by multiplexing (mux), meaning that only a subset of nanopores is actively sequencing at any given time. Each channel is linked to four wells (or mux groups), but only one well per channel is active at any given moment. A mux scan can identify the most suitable nanopores for continued sequencing by testing multiple mux groups (i.e. groups of nanopores) in different channels [38, 39]. During a mux scan, each well is briefly activated to assess the signal quality and classify wells based on pore availability and activity. The scan can detect issues such as blocked pores or unavailable wells and selects the best-performing well for each channel. If needed, MinKNOW will switch to a different well to reduce the inactive time and maximize data output. Note that a mux scan interrupts sequencing of the current reads, so the interval between scans should be adjusted when aiming for very long reads.

The default mux scan is performed every 1.5 h, after which the sequencing continues within the channel, and the current pore can also be directly re-selected. The interval for the mux scan can be set in the MinKNOW software at the start of each sequencing run. This process helps to optimize the sequencing run by ensuring that only high-quality pores are actively sequencing and can increase the overall lifetime of the flow cell.

In addition, the flow cell can be washed during sequencing to unblock and reactivate pores that were unavailable before. For washing the flow cell, the sequencing run needs to be paused or stopped. Then, the flow cell is incubated with nuclease to digest the loaded library. Afterwards, the flow cell can be loaded again with the same or another sample, and the sequencing can continue.

For more details, we refer the reader to the nanopore documentation (www.nanoporetech.com/document/hardware).

## Data characterization and description

This perspective draws its conclusions from an extensive and diverse dataset encompassing over 300 nanopore sequencing runs. Among these, a specific focus is placed on 241 sequencing runs conducted using R9 and R10 flowcells on the MinION and GridION sequencing devices. This dataset offers robust insights into sequencing performance across various conditions. The study also acknowledges instances of sequencing failures and runs conducted on earlier flow cell types (e.g. flongles or R8 flow cells), although these are not included in the accompanying statistical analyses.

The 241 sequencing runs we focus on (see Supplementary Table S1) include 207 DNA sequencing runs and 34 direct RNA sequencing runs. These runs span a wide range of organisms, including 33 viruses, 45 bacteria, 5 protists, 2 plants, 47 insects, 17 mice, 38 humans, 51 metagenomic, and 3 synthetic samples. The amount of input material loaded onto the flow cells ranged from 42 ng to 1440 ng of DNA/RNA. All the RNA runs described in the current study are from direct sequencing of RNA; complementary DNA (cDNA) was not examined.

The output of sequencing runs was assessed based on several metrics: (i) The sequencing yield, defined as the overall amount of sequenced bases, depends on the runtime of the flow cell. We decided to compare the total number of bases produced after 12 h. We chose 12 h because some runs included in this study were stopped after that time to wash the flow cell. Referred to as EB12 in this study and estimated by MinKNOW, EB12 ranged from $7 \cdot 10^6$ to $28 \cdot 10^{9}$ nucleotides. (ii) The median read length, calculated after basecalling, varied significantly, ranging from 419 to 23 746 bases. We chose to calculate the median instead of the mean, as it is more robust against outliers. (iii) Another key metric was the pore half time of the flow cells, defined as the duration of sequencing until only half of the initially active pores remained active. This value ranged from 1 to 59 h, highlighting variations in flow cell durability and performance across experiments. Due to artifacts observed in some flow cells, we manually re-evaluated the pore half time of 22 flow cells, as shown in Supplementary Table S3. (iv) Other flow cell parameters evaluated in this study include the number of active channels, active pores, and the age of the flow cells. The number of active channels, as reported in ONT run reports, ranged from 93 to 512 out of a possible 512 channels. (v) Similarly, the number of active pores varied between 749 and 1984 out of a total of 2048 available pores per run. As the number of active pores can increase shortly after starting sequencing, we used the maximum number of active pores during the first 10 min of each run. (vi) The flow cell age was calculated from the time of their arrival at the laboratory, as the production dates were not accessible. We analyzed flow cells that were used between 2 days and 249 days after their arrival, with ONT’s recommended maximum shelf life being 90 days when stored at 2–8°C. (vii) Last, we investigated how the amount of loaded library (in ng) affected the sequencing yield.

The following analyses provide deeper insights into flow cell performance and durability and have been conducted using custom scripts. Only unwashed sequencing runs were analyzed for the main figures. Runs that included wash steps were analyzed separately. We also tried to address the question, if short reads are sequenced first, which is topic of interest to the community. However, our results were inconsistent in this respect, making it difficult to draw a clear conclusion.

## Challenges in library preparation for ONT sequencing

Library preparation for ONT sequencing involves multiple steps. For instance, DNA library preparation using the Ligation Sequencing DNA V14 kit (SQK-LSK114) entails DNA repair and end-preparation, followed by adapter ligation and clean-up, before priming and loading the prepared library onto the flow cell. An alternative, if short preparation times are a main focus, is the Rapid Sequencing kit V14 (SQK-RAD114), which reduces the preparation time to as little as 10 min at the cost of sequencing accuracy. For DNA, multiplexing is possible [40] using the Native Barcoding Kit 96 V14 (SQK-NBD114.96).

Similarly, library preparation for direct RNA sequencing (SQK-RNA004) begins with adapter ligation of the reverse transcriptase adapter, followed by a reverse transcription reaction to stabilize the single RNA strand, and concludes with the ligation of the RNA ligation adapter, priming, and flow cell loading. Each stage of these protocols requires multiple handling and washing steps, such as pipetting, mixing, and centrifugation, which introduce challenges in maintaining nucleic acid length and achieving consistent yields.

### Obtaining long reads

To obtain the longest possible DNA or RNA reads, it is crucial to minimize any shearing of the nucleic acids. To avoid shearing during library preparation, it is important to reduce the suction force when pipetting. This can be minimized by using cut tips and pipetting slowly, as recommended by Prall et al. [41]. Additionally, vortexing is considered too harsh for nucleic acids, as it often results in shorter fragments, so it is advised to use gentle tapping for mixing instead [42].

Many DNA/RNA isolation kits, particularly those using bead-beating- or column-based methods, are known to shear and fragment DNA nucleic acids [43, 44]. We recommend the classic phenol-chloroform extraction method, as it preserves longer fragments. Recently, there has been a growing interest in high molecular weight DNA extraction techniques. Innovations in this area include the surface topography of silica lamellae and new solid-phase methods introduced by different brands, which aim to improve yield and ease of handling. These kits are often more user-friendly but typically come at a higher reagent cost [45, 46].

In our analysis of DNA and RNA sequencing reads, we mainly used slow and gentle phenol-chloroform extraction to avoid nucleic acid shearing, to maximize read length, and for RNA to include small non-coding RNAs ($<$200 nucleotides), which are lost in many column-based methods [47]. We achieved notably long reads in both DNA and RNA samples. The longest DNA read obtained was 1.4 Mb, which came from a metagenomic sample (sample ID: 117.1) and was classified as *Homo sapiens* using kraken2 [48] and had a GC content of 41.4%. While nanopore sequencing is generally more robust in handling GC-rich regions than polymerase chain reaction (PCR)-dependent platforms, high GC content can still affect read quality, base-calling accuracy, and sequencing coverage [49, 50]. For RNA, the longest read was 175 kb from sample ID 103.1, which was classified as bacterial with a GC content of 68.6%.

### Adjustment of loading amount

#### Loading dependent on pore availability

**Figure 2.**
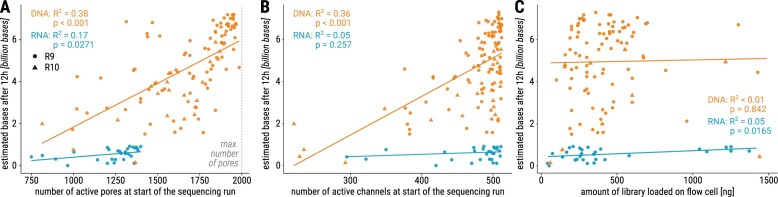
(**A**) The number of active pores (maximum number of active pores during the first 10 min) at the beginning of the sequencing run seems to correlate with EB12. Note the generally lower number of active pores for RNA runs. (**B**) The number of active channels at the beginning of the sequencing run has a weaker association with EB12. Although a high yield is only achievable when many channels are active, starting with many active channels does not automatically guarantee a high EB12. For DNA, a correlation can be seen between the number of estimated bases after 12 h (EB12) and the number of pores and channels at the sequencing start, respectively. For RNA, a relatively weak trend can be seen only for the number of active pores. (**C**) For DNA, the number of estimated bases after 12 h is independent of the amount of starting material loaded onto the flow cell (*R*^2 ^< 0.01, *P* = .842). For RNA, a relatively weak trend shows increased amount of estimated bases after 12 h with increased amount of starting material loaded onto the flow cell (*R*^2^ = 0.19, *P* = .0165). NB, the regression line and *R*^2^ values are shown from linear regression, which resulted in the higher AIC values for DNA compared to second degree polynomial and exponential regression, see Supplementary Table S4.

While it may seem intuitive to increase the amount of DNA/RNA loaded to enhance data yield, there is a threshold beyond which additional loading may not yield proportional benefits (https://nanoporetech.com/document/chemistry-technical-document). This is particularly true due to saturation effects caused by a limited number of pores on the flow cell. The quantity of data generated is directly proportional to the initial number of active pores (Fig. 2A) and channels (Fig. 2B). Linear regression showed a positive correlation between the initial number of active pores and EB12 (*R*^2^ = 0.38, *P*$<$ .001) and between the initial number of active channels and EB12 for DNA (*R*^2^ = 0.36, *P*$<$ .001). For RNA, we observed a weak positive correlation between the initial number of active pores and EB12 (*R*^2^ = 0.17, *P* = .0271), but no significant correlation between the initial number of active channels and EB12 (*R*^2^ = 0.05, *P* = .257).

Our experiments demonstrated that for flow cells with at least 1500 active pores, a DNA load of 350 ng produced reasonable sequencing output. Based on our experience, we found that loading 300 ng of DNA worked well for flow cells with ~1200 active pores, while ~250 ng was suitable for those with ~800 active pores, as detailed in Supplementary Table S1.

In most ONT protocols, the loading amount is recommended in ng or μg; however, especially for genomic DNA (gDNA), an amount in fmol is also given. For first-time use of new preparation methods or sample types, we would recommend to check the length of prepared nucleic acids and calculate the amount in fmol to ensure it falls in the range of recommendations of ONT—and always check the actual protocols as these recommendations change with new chemistry, flow cells or updated protocols. Please note: 1 $\mu$g DNA of 8 kb fragments are 200 fmol; 5 kb are ~300 fmol; 50 kb are 30 fmol.

In our experience, when working with limited amounts of input DNA or RNA, using flow cells with a reduced number of active pores at the start of the sequencing run is sufficient for downstream analyses. However, as expected, lower input material consistently resulted in a reduced number of sequenced reads.

As expected, the library amount [ng] did not influence EB12 for the DNA samples (*R*^2^  $<$ 0.01, *P* = .842), since early in the sequencing run, throughput is limited by the number of active channels, not library availability (see Fig. 2C and Supplementary Table S5). This might change during sequencing, when library is consumed and active pores are still available. A relatively weak trend towards higher EB12 was observed when loading more starting material for RNA though (*R*^2^ = 0.19, *P* = .0165). Note that R10 flow cells exhibit similar behavior in this regard as the discontinued R9 flow cells.

Instead, having extra input material available is advantageous when employing repeated washing of the flow cell, as it helps to extend overall sequencing yield. Specifically, when planning to wash a flow cell, we recommend splitting the library for the number of planned sequencing runs in advance (see details in Section Washing and reusing flow cells).

#### Minimal DNA loads for flow cells

For optimal results in DNA sequencing, ONT recommends to start the library preparation with 1000 ng (equivalent to 100–200 fmol; see Section Loading dependent on pore availability) of total DNA (SQK-LSK114 DNA library preparation kit). Following library preparation, ~50% of the DNA is typically available for loading into the flow cell.

For samples with limited amount of DNA that cannot be amplified, the following methods have yielded successful results: Although ONT’s instructions recommend a minimum of 100 ng, one study showed that without special treatment, 1 ng of DNA (prior to library preparation) yielded 6118 reads with an N50 of 3907 bases [51]. This result indicates good quality; however, reduced input correlates with decreased output. The carrier sequencing method allows for the sequencing of very small amounts of target DNA by combining it with a larger quantity of non-target “carrier” DNA. This technique successfully detected as little as 0.2 ng of target *Bacillus subtilis* DNA combined with 1000 ng of carrier Lambda phage DNA, producing high-quality data without amplification [52]. The 10× Genomics Chromium Controller is an advanced microfluidics-based platform that enables high-throughput single-cell and spatial analysis of DNA, RNA, and proteins. Cells processed by the Chromium Controller will result in barcoded GEMs (Gel Beads in Emulsion), which can be used in downstream applications, like nanopore sequencing, to enhance the analysis of low-input samples [53–55]. This technology allows for the sequencing of gDNA in quantities as low as 50 pg.

In our study, when loading as low as 30 ng DNA (see Section Loading dependent on pore availability) onto a flow cell (435 ng before library preparation, sample ID 67.6) we successfully obtained 648 Mb sequencing data. In another run we loaded 50 ng onto the flow cell (sample ID 129.2), which resulted in 1.5 Gb data output. Simon *et al.* reported the successful use of 50 ng in metagenomic samples with eight species [56].

#### Minimal RNA load for flow cells

RNA sequencing requires poly-adenylated 3′ ends (poly-A tails), which serve as consistent binding sites for ligating adapter molecules to create a continuous RNA–adapter complex that can pass through the nanopore (see Section Adapter ligation). Ribosomal RNA (rRNA), which lacks poly-A tails, makes up the majority of cellular RNA; for instance, *Escherichia coli* cells are estimated to contain around 85 % rRNA [57]. rRNA is naturally excluded when using a poly-A-based sequencing approach. For optimal RNA sequencing, we advise the user to always check the latest protocol version on the ONT website. Sequencing in this study was performed with RNA002, where input recommendations were 500 ng of total RNA or 50 ng of poly(A)-tailed RNA (which is in agreement with the ratio of rRNAs in the cell). However, this changed to 300 ng of poly(A) tailed RNA or 1 ug of total RNA with the current RNA004 protocol and chemistry (version DRS 9195 v4 revF 11Dec2024).

Many RNA molecules, including certain messenger RNAs (e.g. histone-coding) and many transcripts (like ncRNAs and non-canonical microRNAs), lack poly-A tails [58, 59]. To sequence all RNAs except rRNAs, it is recommended to add poly-A tails to the sample and perform an rRNA depletion step following the poly-A addition by polyA polymerase [60]. A method for total RNA sequencing with only 10 ng input material has been reported [61]. In our study, 42 ng of RNA (sample ID 152.1) produced 157 Mb of data.

### Using external control sequences

The use of external control sequences (spike-ins) is a critical aspect of controlling library preparation and benchmarking the sequencing process in nanopore workflows. External controls consist of nucleic acid molecules with known sequences, which are introduced into a sample in precise amounts. These molecules undergo all the same procedural steps as the sample’s endogenous nucleic acids and are only distinguished during the final stage of sequencing data analysis. Both the DNA ligation sequencing kit (SQK-LSK114) and the direct RNA sequencing kit (SQK-RNA004) contain positive control strands. The DNA Control Strand (DCS, Supplementary Fig. S1) is a 3.6 kb standard amplicon that maps to the 3′ end of the Lambda virus genome. It is provided at a concentration of 10 ng/μl (according to ONT Live support asking “What is DCS?,” https://nanoporetech.com, accessed 12.12.2024, see Supplementary Fig. S2).

The RNA Calibrate Strand (RCS) is derived from the *Saccharomyces* genome, specifically the Enolase II gene (ENO2, YHR174W). It is supplied at a concentration of 15 ng/μl (according to ONT Live support asking “What is RCS?,” https://nanoporetech.com, accessed 12.12.2024, see Supplementary Fig. S3). Both DCS and RCS are utilized as positive controls for the library preparation and sequencing processes; and therefore are not used for normalization of sequencing results. In our experiments, we detected ~1 % of DCS/RCS sequences in our samples.

In addition to the ONT kit-supplied control sequences, the emergence of various RNA-seq platforms and protocols has highlighted the need for versatile RNA spike-in controls. These controls can be incorporated and processed alongside actual samples, allowing researchers to monitor and compare essential performance metrics such as sensitivity, input-output correlation, and the detection and quantification of transcript variants. Spike-In RNA Variants (SIRVs) are specifically designed synthetic RNA molecules that replicate key features of transcriptome complexity. Added in very small quantities before library preparation, SIRVs can be processed identically to endogenous RNA, ensuring accurate assessment of sequencing performance.

### Adapter ligation

In adapter ligation, the final library preparation step for nanopore sequencing, adapters are attached to sample molecules. These adapters contain motor proteins that guide DNA or RNA into the nanopore and regulate sequencing speed, making ligation efficiency crucial for optimal output. The exact concentration of the adapters in the mixes is proprietary; however, it is standardized for reactions involving ~100–200 fmol i.e. approx. 1 μg of DNA (see Section Loading dependent on pore availability), 50 ng for poly(A)-tailed RNA, and 500 ng for total RNA.

New England Biolabs (NEB) generally recommends maintaining a 10:1 adapter-to-sample ratio to ensure efficient ligation without excessive free adapters. According to ONT (Chapter 43, “Troubleshooting your run from the pore activity plots”), high adapter-to-sample proportions generally do not hinder sequencing as long as the majority of pores are actively sequencing.

### Amplicon sequencing

Amplicon sequencing is a common strategy to enrich low concentrations of nucleic acid target material from complex samples for subsequent sequencing [62–64], including vector sequence confirmation, targeted sequencing of full-length cDNAs, confirmation of disease-causing variants, and virus sequencing. Here, we focus on viral sequencing as one of many examples for amplicon sequencing [65–67]. Amplicon sequencing enriches specific DNA regions by amplifying them via PCR with specific primers. The resulting PCR products, or ‘amplicons,’ are then sequenced to provide high-resolution information on the targeted regions. A key example of amplicon sequencing is its application in viruses, such as SARS-CoV-2 during COVID-19, for cost-effective sequencing and genomic surveillance [68]. ONT has specific advantages in sequencing long amplicons without fragmentation, e.g. to retain information of co-occurring mutations on single amplicons supporting virus variant deconvolution and lineage assignment [69].

In nanopore sequencing, amplicons should be mixed in equal nanomolar concentrations [70–72]. When working with amplicons of the same size, mass concentrations (nanograms per microliter) can be used instead of molar concentration. Due to the specific target enrichment, amplicon sequencing can ensure sufficient sequencing depth to reliably detect genetic mutations, supporting detailed genomic analysis in fields requiring accurate variant detection.

**Figure 3.**
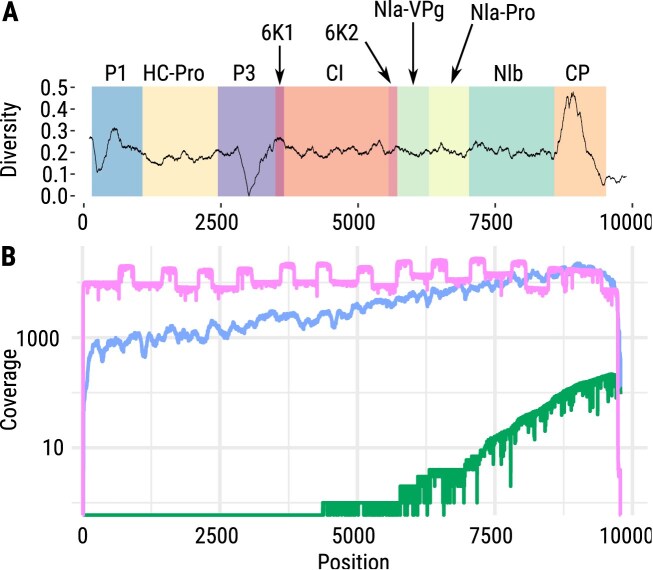
(**A**) Genetic diversity shown for each position in the PPV genome across 109 PPV isolates of all known PPV strains indicates reduced genetic diversity in the P3 gene region (purple) and enhanced genetic diversity in the genetic region encoding for the capsid protein (CP, orange). (**B**) Coverage comparison of three sequencing methods: direct RNA sequencing using nanopore (green), Illumina shotgun RNA-seq (blue), and nanopore-based PCR amplicon cDNA sequencing (pink) at the example of PPV. A characteristic feature of amplicon sequencing is the clear visibility of higher coverage regions due to the overlapping amplicon design and specific enrichment.

Amplicon sequencing can be an effective approach to explore entire virus genomes with low viral titers. While a lot of work was done on SARS-CoV-2, amplicon designs are increasingly being explored for other viruses, such as plum pox virus (PPV). High-quality assemblies require overlapping amplicons, as accurate strain identification is crucial for diverse pathogens. In some protocols, however, such as the widely used ARTIC protocol (https://github.com/artic-network/artic-ncov2019) for SARS-CoV-2 sequencing, smaller amplicons of 392 bp are employed, with an overlap of 90 bp. In our study of SARS-CoV-2, we achieved high-quality results using longer amplicons of ~1200 bp, with an overlap of 117 bp between consecutive fragments [73]. We demonstrate an application of amplicon sequencing for the PPV genome, which exhibits substantial genetic diversity across strains (Fig. 3A). We gained a uniform distribution of coverage across the entire genome (see Fig. 3B), demonstrating the effectiveness of well-designed amplicons for comprehensive genomic representation. Monitoring the amplicon coverage is essential, as the aforementioned diversity may result in poor amplicon performance due to insufficient primer binding in mutated regions.

### Direct RNA sequencing

The direct RNA sequencing protocol provides an approach for sequencing RNA molecules directly without the need for converting them into cDNA [14]. This method allows for sequencing RNA in its native form, thereby preserving RNA modifications, without amplification, and potentially capturing full-length RNA transcripts. An optional library preparation step involves synthesizing a cDNA strand from the RNA template through reverse transcription. Formation of RNA:cDNA hybrids can increase throughput and improve read quality by resolving complex RNA secondary structures, and can result in more consistent sequencing data [74].

However, this approach has two downsides: (i) prolonged incubation of RNA at high temperatures with divalent cations during cDNA synthesis can cause RNA degradation [75, 76]; (ii) common reverse transcriptases may cleave RNA strands. This can reduce the availability of full-length RNA and impact the efficiency and accuracy of direct RNA sequencing [77, 78].

A distinctive feature of ONT’s direct RNA sequencing is its ability to detect RNA modifications. However, the bioinformatics analysis of these modifications is still underdeveloped. In this work, we focus intensively on this aspect, see Section Hidden treasures in raw data.

## Technical challenges in nanopore sequencing

### Maximizing sequencing yield

Sequencing yield, also referred to as throughput, represents the total amount of data generated during a sequencing run. According to Wang *et al.* [2], the expected sequencing yield of a flow cell primarily depends on three factors: the number of active nanopores, the DNA/RNA translocation speed through the nanopore, and the running time. If the translocation speed is too fast, the system may struggle to differentiate between nucleotides, resulting in inaccurate sequencing. Conversely, if the translocation speed is too slow, throughput is reduced. The translocation speed is largely dependent on the motor protein used. For DNA, it typically operates at around 400 nt per second [2], while for RNA, the speed is slower, at ~130 nt per second, as stated by ONT. This speed can be monitored in real time if live basecalling is enabled, although it can only be controlled indirectly. For example, increasing the temperature may accelerate the translocation speed, whereas overloading the flow cell might lead to a reduction in speed.

**Figure 4.**
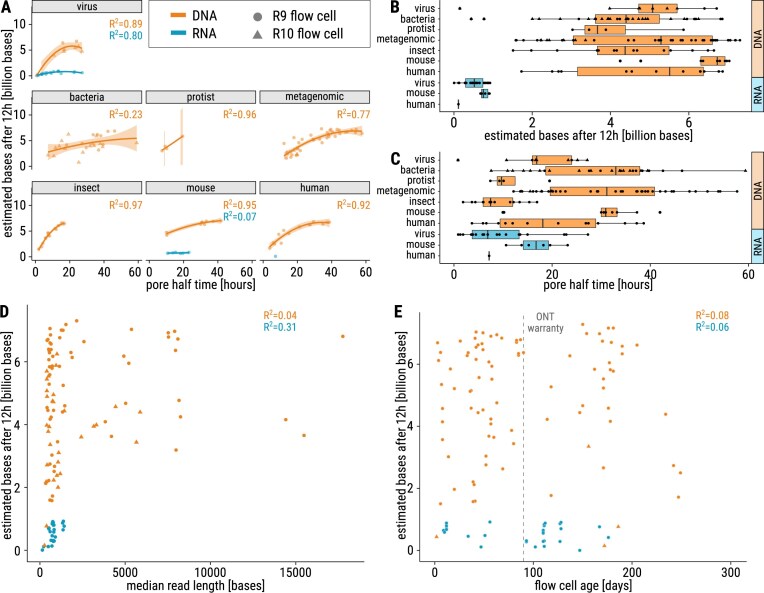
Maximizing sequencing yield. Sequencing yield from R9 (dots) and R10 (triangle) for DNA (orange) and RNA (blue) samples from a variety of species. Differences between DNA and RNA are evident in all plots, with a lower sequencing yield for RNA in general. Note that the lower RNA yield is dependent on the slower sequencing speed [2] and the generally low number of active starting pores during RNA runs. (**A**) Quadratic regression shows a significant correlation of pore half time on estimated number of bases after 12 h of sequencing (EB12) of DNA for virus, metagenomic, insect, mouse, and human samples (*R*^2^ > 0.7 and *P*-value < .01), but not for bacteria and protist (*P*-value > .05). The bacteria samples show high variability, and protist sample size is very low. For RNA, a significant correlation was found in the virus samples (*R*^2^ = 0.8 and *P*-value < .01). Regarding R9 and R10 data separately, the correlation for bacteria slightly increases (R9: *R*^2^ = 0.39, R10: *R*^2^ = 0.11), but still remains weaker than for the other sample types. For the other sample types, the correlation does not change when separating R9 and R10 flow cell data. (**B**) The EB12 yield is consistent across all sample types and flow cell type. (**C**) In contrast to EB12, the distribution of pore half time differs strongly between the sample types. Insect samples have a lower pore half time than other samples. Metagenomic and bacteria samples show a high variance. Data shown in panels (B) and (C) indicate differences between R9 and R10 data for metagenomic samples, see Supplementary Fig. S8. (**D**) The median read length does not influence EB12 (nor the amount of estimated bases after 24 and 36 h, see Supplementary Fig. S9) for DNA. For RNA, a weak positive correlation is visible, which is slightly stronger after 24 and 36 h, see Supplementary Fig. S9. But note that there are only a few data points for RNA after 24 and 36 h, and the amount of starting pores for RNA was generally lower. (**E**) The age of the flow cell (number of days from arrival to usage) does not affect EB12, although our data for R10 flow cells are limited.

To analyze sequencing yield, we explored over 300 ONT sequencing experiments across a variety of species, see Section Data characterization and description, Supplementary Table S1. We observed significant variation in sequencing yield and investigated potential reasons for this yield disparity (Figs 2 and 4). We first investigated which of the measured variables has an influence on EB12. A multiple linear regression analysis revealed that EB12 was significantly predicted by the number of active pores at the start of the sequencing run, pore half time, sequencing material (DNA, RNA), sample type, and flow cell version (R9, R10), see Table 1.

**Table 1.** Multiple linear regression analysis revealed that EB12 was significantly influenced by “starting pores,” “pore half time,” “sequencing material,” and further influenced by “sample type” and “flow cell version”

	Estimate	Std. Error	*t*-value	Pr($> |$t$|$)	Sig.
(Intercept)	$-5.13\times 10^{8}$	9.49$\times 10^{8}$	$-$ 0.541	0.590140	
Starting pores	3.04$\times 10^{6}$	8.59$\times 10^{5}$	3.541	0.000736	***
Starting channels	$-6.74\times 10^{5}$	3.28$\times 10^{6}$	$-$ 0.206	0.837636	
Pore half time	1.14$\times 10^{6}$	1.74$\times 10^{5}$	6.532	1.09$\times 10^{-8}$	***
Library loaded	$-1.50\times 10^{5}$	4.11$\times 10^{5}$	$-$ 0.365	0.716424	
Sample - human	$-4.65\times 10^{8}$	5.52$\times 10^{8}$	$-$ 0.843	0.402119	
Sample - insect	$-6.22\times 10^{8}$	7.30$\times 10^{8}$	$-$ 0.853	0.396922	
Sample - metagenomic	$-1.19\times 10^{9}$	4.39$\times 10^{8}$	$-$ 2.714	0.008470	**
Sample - mouse	$-4.02\times 10^{8}$	5.09$\times 10^{8}$	$-$ 0.791	0.431852	
Sample - protist	$-7.67\times 10^{8}$	6.71$\times 10^{8}$	$-$ 1.143	0.257067	
Sample - virus	$-5.04\times 10^{7}$	5.91$\times 10^{8}$	$-$ 0.085	0.932298	
RNA/DNA - RNA	$-2.94\times 10^{9}$	6.25$\times 10^{8}$	$-$ 4.698	1.38$\times 10^{-5}$	***
Buffer - SFB	$-1.69\times 10^{8}$	4.25$\times 10^{8}$	$-$ 0.397	0.692883	
Buffer - RNA	NA	NA	NA	NA	
Fc version - R10	$-1.64\times 10^{9}$	5.52$\times 10^{8}$	$-$ 2.965	0.004208	**
Read length	1.04$\times 10^{4}$	4.15$\times 10^{4}$	0.250	0.803204	

For the categorical variables one level (first mentioned) is taken as default, and changes to this level are analyzed. These are “sample type” (sample): bacteria, human, insect, metagenomic, mouse, protist, virus; “sequencing material”: DNA, RNA; “buffer”: LFB (DNA), SFB (DNA), RNA; and “flow cell version” (Fc version): R9, R10. The following significance codes were used: *** < 0.001, ** < 0.01, * < 0.05. All variables were analyzed also in a single linear regression, see Supplementary Table S5.

Next, we analyzed the associations of the single variables in more detail, starting with the investigation of EB12 and the pore half time, which refers to the duration after which half of the nanopores in a sequencing run become inactive. Quadratic regression analysis revealed a significant correlation between pore half time and EB12 for DNA within all sample type groups except bacteria and protists (Fig. 4A) consistent for both R9 and R10 flow cells. This correlation remains significant even when normalizing EB12 for the number of starting pores, as shown in Supplementary Fig. S5. As expected, a slower degradation rate of the flow cell leads to a higher sequencing yield.

Next, we analyzed the impact of sample type on EB12. The DNA sequencing yields in our data are generally higher than those for RNA, as shown in Fig. 4B, likely due to increased translocation speed and higher number of starting pores. Although DNA mouse samples had a slightly higher EB12 than other sample types with *P*-values of .04 or below (except *P* = .067 for viruses), see Supplementary Fig. S6, it appears that after 12 h of sequencing, the sequencing yield was roughly similar, regardless of the sample type group. However, multiple linear regression analysis revealed that sample type, especially metagenomic samples, significantly influences EB12, indicating subtle but systematic differences between sample categories.

In contrast, the pore half time seemed to vary significantly depending on the type of sample being sequenced (Fig. 4C, with a median pairwise *P*-value of .011, see Supplementary Fig. S7). For DNA samples, for instance, we found that insect and protist samples tended to have a much shorter pore half time than mouse, metagenomic, or bacterial samples. Furthermore, flow cells used for bacterial, metagenomic, and human samples exhibited high variability in pore half time. The flow cell pore half time appeared to be influenced not only by DNA/RNA characteristics but also by the sample type. We assume these differences might originate from factors such as the quality and purity of DNA/RNA, the extraction method, the sequencing kit used, or the cell type composition. Further research is needed to investigate those factors.

Despite variations in pore half time, the distribution of EB12 appears more consistent across sample type groups, even though the influence of (metagenomic) sample type on EB12 was demonstrated in multiple regression analysis in Table 1. Notably, insect and protist samples, which exhibit shorter pore half time, did not stand out as outliers regarding EB12. To conclude, the sequencing yield produced within the first 12 h (EB12) varied less between the sample types in contrast to pore half time. When comparing data from R9 and R10 flow cells, as shown in Supplementary Fig. S8, a significant reduction in EB12 was observed when using R10 flow cells for bacteria, metagenomic, and viral samples (i.e. all sample type groups for which data from both R9 and R10 flow cells were available). These findings align well with the influence of flow cell type on EB12 identified in the multiple regression analysis in Table 1. Additionally, we analyzed the association of the buffer type (LFB/SFB) with EB12. Though a *t*-test comparing EB12 for SFB and LFB on R9 flow cells shows significant results (see Supplementary Fig. S4), multiple regression analysis revealed confounding effects by the other variables (see Table 1), as the amount of active pores at the start of the sequencing run. Taken together, after 12 h, the sequencing output depended on the number of active pores at the start of the sequencing run (Fig. 2) but remained relatively consistent regardless of sample type and flow cell type. The sample type seemed to be related to the degradation rate of the flow cell.

Most runs had a median read length below 3 kb, with EB12 values ranging from 0 to nearly 8 Gb and showing a fairly even distribution. The median read length had no impact on EB12 (see Fig. 4D), nor the amount of estimated bases after 24 and 36 h (see Supplementary Fig. S9). Here again, we see a clear distinction between DNA and RNA EB12 values. The R10 median read lengths aligned with those of R9 data.

We then turned our attention to the properties of the flow cells themselves. The age of the flow cell (the time between delivery and use) did not significantly affect EB12. Surprisingly, flow cells over 200 days old (Fig. 4E) performed similarly to younger ones, although exceeding the ONT warranty period of three months by a factor of two. The oldest flow cell we tested was 249 days old, performing normally (1080 pores, 3.1 Gb output), see Supplementary Table S1, ID 172.1.

In summary, a high EB12 is only achievable when many pores/channels are active at the beginning of the sequencing run (Fig. 2A and B). However, starting with many active channels does not automatically guarantee a high EB12. On the other hand, as expected, the number of active pores at the beginning of a sequencing run influence EB12, particularly when sequencing DNA (Fig. 2A).

### Washing and reusing flow cells

Flow cells naturally degrade with use as active pores decrease, often due to blocking during sequencing. Blocked pores shift from “single pore” to “unavailable” in MinKNOW. While ONT acknowledges pore blocking, the reasons remain unclear. Pores can be unblocked using voltage reversal during mux scans or by washing the flow cell.

**Figure 5.**
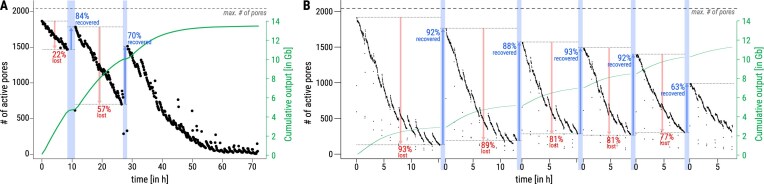
Influence of washing the flow cell on the amount of active pores. (**A**) A flowcell which has been paused (blue bars) two times for washing. (**B**) A flowcell which has been stopped (blue bars) five times for washing.

Washing is performed with ONT’s “Flow Cell Wash Kit” (EXP-WSH003, EXP-WSH004) to digest and remove loaded genetic material, which free blocked pores. The washing process involves flushing the flow cell with the wash buffer provided in the Wash Kit to remove residual sequencing reagents. This is done by slowly injecting the washing solution through the priming port and then carefully removing it after 1 h incubation to maintain pore viability. It is crucial to avoid introducing air bubbles. The cost of washing is minimal, ~€16 per washing step, as the Wash Kit EXP-WSH004 costs €95 for six washing steps (23 October 2024). The sequencing run can be paused or stopped before washing: pausing allows continuation in the same file, while stopping generates a new file for subsequent sequencing. After washing, the flow cell can be reused for sequencing a new sample, but the previous run must be stopped rather than paused. Washed flow cells can also be stored for future use. However, they will not perform as well as new ones; the number of active pores will be lower than initially but higher than before washing (see Fig. 5).

Washing can also be employed during a single sequencing run to increase sequencing yield by reactivating blocked pores. We identified three key factors to consider before sequencing: (i) potential washing steps, (ii) optimal washing timing, and (iii) available sample material. We investigated the impact of washing flow cells on pore recovery by analyzing 34 flow cells that had undergone 1–6 washes, totaling 58 washing steps, see Supplementary Table S1. Exemplarily, Fig. 5 depicts two such washing steps. Statistics of these two flow cells are displayed in Supplementary Table S6. In Fig. 5A, a human DNA sample (ID 111.1) was washed twice during sequencing (at 10 and 30 h), pausing the run (blue bars). Active pores decreased over time (red arrows) but increased after each wash (blue arrows). In Fig. 5B, a cricket DNA sample (ID 124.1–124.6) was washed five times during sequencing. A mean loss of 84.2% of active pores could be compensated by recovering on average 85.6% of those pores. ONT recommends washing and reusing a flow cell up to four times. Our findings show that flow cells can be washed at least five times and still produce data.

A key factor for high sequencing yield is determining the appropriate time point for washing, as yield depends on the number of active pores, see Fig. 2A. It appears that washing does not cause pores to block more quickly. Therefore, we recommend monitoring the cumulative output in MinKNOW: when the curve flattens (Fig. 5A, green line), we advise washing.

To ensure sufficient material for reloading, we recommend splitting the sample before starting the sequencing run if washing is planned to increase yield. For instance, when planning two wash steps, we recommend allocating 50%, 30%, and 20% of the total library for the initial run and subsequent washes, respectively. This decreasing allocation accounts for the reduction in active pores after each wash. Adding excess material beyond the capacity of available pores will not further increase sequencing yield (see Figs 2C and 5).

### Adaptive sampling

Adaptive sampling allows for real-time selection or rejection of DNA fragments based on their sequence content, enabling targeted enrichment or depletion of specific genomic regions, organisms, or sequence types during a sequencing run (https://nanoporetech.com/document/adaptive-sampling) [79]. For example, adaptive sampling can enrich gene panels, CpG islands, or exomes [80], eliminate unwanted sequences such as highly repetitive regions or contaminants, and target specific organisms or deplete host in metagenomic studies [26, 81, 82]. This approach enhances coverage of targeted genes or regions without requiring complex library preparation steps like amplification or hybridization probes.

**Figure 6.**
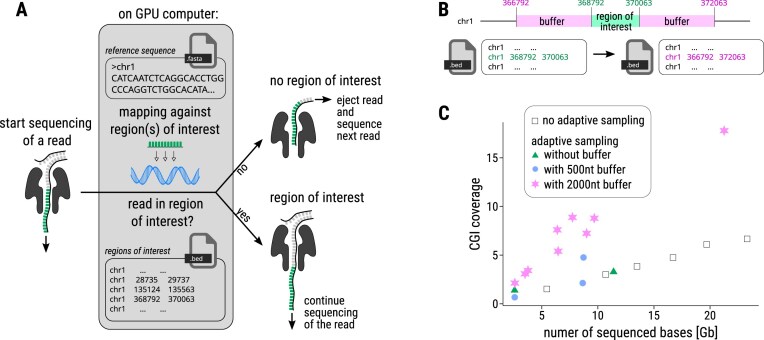
**A**) The basic principle of adaptive sampling: For each read, the first ~400 bases are mapped to a given reference, and sequencing continues only if they match a region of interest [26]. About 450 bases are sequenced per second for R9 nanopore flow cells [2]. Rejecting a read takes ~0.5 s, with additional time required to capture the next read. (**B**) In order not to miss the reads where the region of interest is not at the beginning of the read, the target region should be defined with an extended buffer (e.g. 2000 nt) on both sides in the configuration .bed file. (**C**) Adaptive sampling was used to enrich over 30 000 CpG islands (CGIs) to increase their coverage. To increase enrichment, each CGI region of interested was extended by a 500 nt buffer region and by a 2000 nt buffer region on both sides.

As DNA molecules pass through a nanopore, the device generates real-time sequence data (Fig. 6A). This requires a GPU and a fast basecalling model for adaptive sampling. The system quickly compares each fragment’s partial sequence (~400 bases, corresponding to one second of data) to a reference genome or user-defined targets. When enriching for regions of interest, the sequencing continues only if the sequence matches the region of interest; otherwise, the DNA fragment is ejected, conserving flow cell resources and improving yield for desired regions. The depletion mode works vice versa.

As the acceptance or rejection of a read is based solely on its first chunk, strands may be rejected, if the region of interest is not located at the beginning of the read. To address this, the target region of interest should be extended by adding a ‘buffer’ on both sites in the configuration file to accommodate sequencing in either direction, as sequencing can initiate from either DNA strand (Fig. 6B). Thus, for adaptive sampling, the buffer describes a certain number of nucleotides by which the region of interest can be extended in the bed file (a text file format used to store genomic regions).

Applying adaptive sampling may yield a higher number of short reads (reducing N50 and median read length) and necessitate re-basecalling with a higher accuracy model post-sequencing to enhance the data quality.

We applied adaptive sampling to enrich CpG islands in the human genome, see Supplementary Table S7. We targeted ~30 000 CpG islands, with an average length of 777 nt (IDs 153.1–178.1). We applied adaptive sampling without adding a buffer region, using a 500 nt buffer region, and a 2000 nt buffer region on both sides of the target regions. We achieved up to 17 × mean sequencing depth on CpG islands, representing a fourfold increase compared to the mean sequencing depth of the whole genome, when applying 2000 nt buffer (Fig. 6C). According to ONT, the theoretical maximum increase in sequencing depth achievable through adaptive sampling is ~5–10-fold (https://nanoporetech.com/document/adaptive-sampling). In summary, adaptive sampling can effectively enhance sequencing depth in specific genomic regions or target species, albeit with increased time requirements for post-processing, such as re-basecalling and filtering out short, unwanted reads.

## Challenges in analyzing ONT data

### Building an ONT pipeline

**Figure 7.**
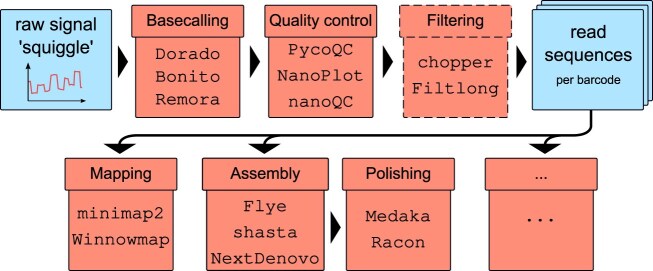
The standard pipeline for nanopore sequencing data involves basecalling of the raw data, followed by quality control (QC) and filtering. The reads can then be analyzed with specialized long-read tools for mapping, polished assembly, or other workflows.

Bioinformatics analysis of ONT sequencing data is based on modular pipelines that can be assembled and utilized effectively, even by users with limited computational backgrounds (Fig. 7). Instead of relying solely on standard, one-size-fits-all pipelines, it is critical to carefully select tools that best align with specific experimental goals and data characteristics. The following pipeline steps are very similar between DNA and RNA sequencing with ONT up to and including the mapping step. Afterwards, the processing steps usually diverge and become more specific [83].

A well-constructed ONT analysis pipeline includes several steps, which can be addressed by different tools:

Basecalling is the transformation of raw signal data, or “squiggles,” into nucleotide sequences using neural networks. Typically, the basecalling tools have a pre-processing step, such as removing the adapters and normalizing individual signals. The following three tools are all provided by ONT. Currently, Dorado (https://github.com/nanoporetech/dorado) is the state-of-the-art software for basecalling on ONT platforms that supports the older fast5 and the new pod5 format, which is a more compact format enabling faster and more efficient file access. Dorado includes basecalling models for all ONT kits, predicts modified bases, and has built-in demultiplexing capabilities for barcode detection from ONT barcoding kits. Bonito (https://github.com/nanoporetech/bonito) is an alternative tool that allows users to train their own neural networks for basecalling. Remora (https://github.com/nanoporetech/remora) adds an additional layer, enabling nucleotide modification calling by training neural networks along the standard bases. Alternative tools for nucleotide modification prediction are discussed in detail below. Basecalling results are typically saved in fastq format for subsequent analysis steps.

After basecalling, QC is essential to evaluate the reliability of the sequencing data and to identify any potential issues in the dataset, such as degradation, contamination, and read length distribution. One of the most comprehensive QC tools for ONT data is pycoQC (https://github.com/a-slide/pycoQC) [84], which provides an interactive html overview that includes information on read quality, read length, sequence coverage, active channels, and quality over sequencing time. Other QC tools, such as NanoPlot (https://github.com/wdecoster/NanoPlot) [85] and nanoQC (https://github.com/wdecoster/nanoQC), provide a restricted functionality, but with a different representation of the data.

Following QC, filtering is often necessary, especially for experiments requiring specific read quality or length thresholds. Filtering tools help to remove low-quality reads, adapters, barcodes, or reads outside the desired range, increasing the overall quality and relevance of the dataset. Chopper (https://github.com/wdecoster/chopper) from the NanoPack suite [85] or Filtlong (https://github.com/rrwick/Filtlong) are widely used for filtering ONT reads. Dorado also provides parameters to trim adapters and barcodes.

Once high-quality reads are obtained, subsequent analyses such as mapping and assembly can be performed using specialized tools designed for long-read data. For read mapping, minimap2 (https://github.com/lh3/minimap2) [86] is widely used. Winnowmap (https://github.com/marbl/Winnowmap) [87] is an alternative mapping tool that has been specifically optimized for complex genomes. It has demonstrated superior performance when mapping both simulated PacBio and ONT data for human genomes [87]. For assembly, Flye is one of the most widely used tools, demonstrating good performance in terms of contig length and error rate in bacteria [88]. It includes polishing and can be told how many iterations of polishing should be performed. Alternative tools, such as Shasta (https://github.com/paoloshasta/shasta) [89], have been shown to perform faster, while NextDenovo (https://github.com/Nextomics/NextDenovo) [90] can generate longer contigs, particularly in the case of mollusks [91]. Error correction (polishing) can be subsequently utilized to refine the assembly. Medaka (https://github.com/nanoporetech/medaka) is aN ONT-specific neural networks, trained on typical ONT errors to correct these errors effectively. Medaka has proven particularly effective in reducing deletion errors [88]. Additionally, Racon (https://github.com/isovic/racon) [92] is a consensus caller using high quality reads from technologies such as Illumina to reduce single nucleotide variants and insertion errors, enhancing the overall accuracy of the assembled data.

### Hidden treasures in raw data

Beyond the standard pipeline of ONT tools, the raw data from ONT sequencing contains information about nucleotide modifications. ONT provides basecalling models to detect specific modifications using the Dorado basecaller, such as 4mC, 5mC, 5hmC, and 6mA for DNA, and m5C, m6A, Ino, psU and 2′-O-me modifications (mC, mG, mA, and mU) for RNA; see Supplementary Table S2.

To detect other modifications, using modification detection tools or analysis of the raw ONT data is needed. Analyzing the raw signal requires significant self-implementation and detailed knowledge. Here, a demonstration of the fundamentals necessary to fully exploit the potential of ONT by using DNA and RNA modification detection as an example is given. This description will pave the way to complement existing tools for nucleotide modification analysis, which are currently designed for only a small fraction of modifications. To achieve this, we focus on processing the raw data, which can be divided into five distinct phases: (i) Accessing raw data: in this initial step, the raw signal data from the nanopore sequencer is imported into the computational analysis. This is particularly important because it serves as the foundation for all subsequent analyses. (ii) Improving Signal Segmentation: is applied to refine the process of identifying individual signals corresponding to nucleotides. Improving segmentation can help to enhance the accuracy of the subsequent basecalling step by ensuring that the software can effectively distinguish between different signal components. (iii) DNA Modification Detection: Once the signal data is well-segmented, this step focuses on identifying specific modifications to DNA (e.g. methylation). Detection of DNA modifications is important for understanding epigenetic regulation and gene expression, providing insights into cellular functions and biological processes. (iv) RNA Modification Detection: Similar to DNA modification detection, this step involves identifying modifications in RNA sequences. RNA modifications can influence gene expression and stability, making their detection crucial for studying transcriptomics and understanding post-transcriptional regulation. (v) Differential Signal Detection: Alternatively to predicting modification types directly, the differences in signal patterns between conditions or samples can be analyzed to reveal a general change of the nucleotide.

#### Preprocessing raw data

**Figure 8.**
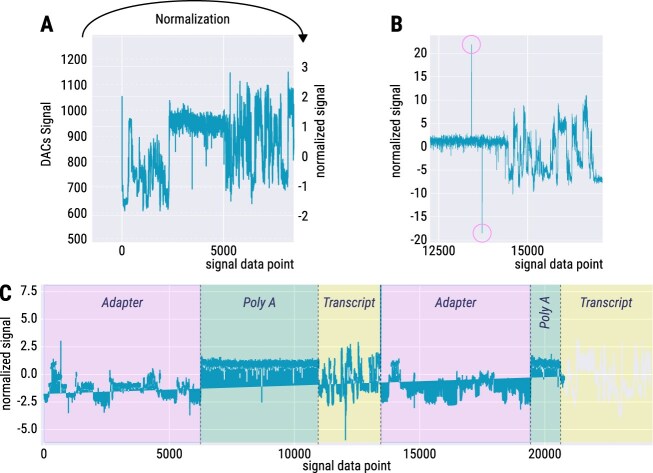
Overview of signal data preprocessing steps. (**A**) To standardize the data, the unnormalized time series (left) are converted (arrow) into the corresponding normalized signal (right, mostly ranging from −4 to 4). (**B**) Detected outliers (pink circles) indicate points that deviate significantly from the expected signal pattern, likely due to sensor errors. (**C**) A single signal can contain more than one read. Here indicated with signal containing three recurring components for “Read 1” and “Read 2” specific to RNA sequencing: “adapter,” “poly A,” and “transcript.”

ONT stores the “squiggles” as integers [93] to save storage space, which can be converted back to pA; see Supplementary Equation S1. Normalization of the raw ONT signals is essential for reliable comparisons within and across sequencing experiments. Signal variations caused by differences in flow cells, nanopores, and sensors can introduce biases (Supplementary Fig. S10), making normalization (Fig. 8A) critical for accurate and reproducible analyses (Supplementary Equation S2). Outliers in ONT signals, as in Fig. 8B, are often caused by faulty sensor measurements and can distort downstream analyses. To ensure data quality, these outliers should be identified and filtered prior to further signal processing. The ONT signal, comprising a continuous series of data points, may occasionally include multiple (adjacent) sequencing reads, as shown in Fig. 8C. The reason for this phenomenon is unresolved. To ensure accurate and reliable downstream analyses, such signals must be split into individual reads using the coordinates provided by Dorado.

The python package read5 (https://github.com/rnajena/read5) is a wrapper that unifies access to fast5, pod5, and slow5 data formats and provides functions for signal conversion (Supplementary Equation S1) and normalization (Supplementary Equation S2). Each of these file formats has its own API using different function names for the same functionality, which often requires to write specific code to handle these inconsistencies.

#### Improving signal segmentation

**Figure 9.**
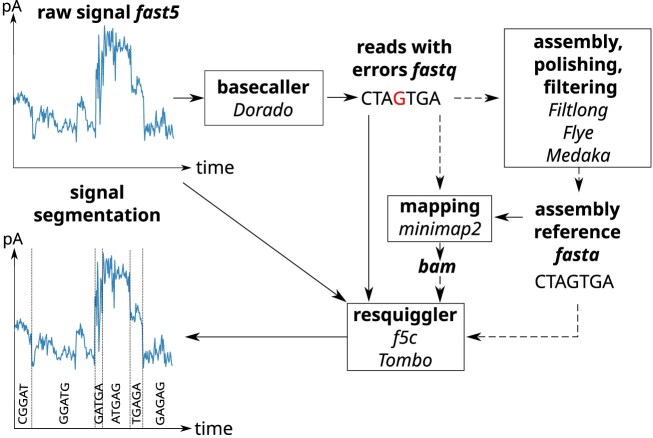
Overview of signal processing and modification calling. The raw ONT signal can be segmented and corrected for basecalling errors by resquigglers using the raw signal and basecalls. Some resquiggling tools also require a reference sequence and a mapping.

The analysis of raw data plays a crucial role for detecting chemical nucleotide modifications. This is particularly relevant for the vast number of around 170 RNA modifications for which no basecalling models currently exist. These modifications can produce characteristic signals when translocating through the nanopore. Analyzing raw signals for modification detection requires improved signal segmentation, known as ‘resquiggling.’ This process involves realigning the raw current signal to the nucleotides, see Fig. 9. This segmentation is crucial because the motor protein that moves the nucleotides through the pore does not operate at a constant speed. The time a nucleotide spends in the pore (‘dwell time’) varies depending on the specific nucleotide [94].

Several tools have been developed to facilitate the resquiggling process and generally require (i) the raw ONT signal (fast5, slow5, or pod5) files and (ii) the basecalls generated by the basecalling process (fastq). Optionally, (iii) a reference sequence (fasta) and (iv) the alignments of the basecalls to the reference (bam) can be added. The most widely used tool f5c [95] is a GPU-accelerated, multi-threaded re-implementation of Nanopolish Eventalign. It offers significant reductions in runtime while maintaining the known output formats. Notably, f5c also supports the newer pores, such as R10 and the new RNA pore (RP4). For instance, a sequencing run of a 32 000 bp Coronavirus genome (sample ID 13.1) with 1.3 Gb across 857 000 reads produced a 181.5 GB output file using Nanopolish Eventalign, which was reduced to 84 GB with f5c. Tombo has not been supported by ONT since 2020 and is compatible only with older single-read fast5 formats. Despite its limitations, Tombo remains in use today for specific applications.

Future developments in resquiggling tools are expected to further enhance compatibility with emerging nanopore technologies and data formats.

#### Detecting DNA modifications

**Figure 10.**
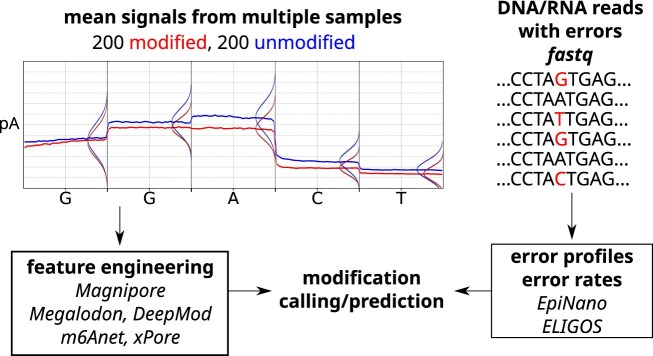
Modified nucleotides have their own characteristic signal. Machine learning models can detect this signal directly or by comparing it to the unmodified counterpart. The red and blue lines represent the mean signal of 200 reads, respectively, while the vertical bulges indicate the signal distribution per base following a normal distribution. Modifications can lead to error patterns in the reads that can be detected by tools.

The direct detection of DNA modifications without amplification or pre-treatment of the DNA is an exciting feature of nanopore sequencing, potentially providing insights into epigenetic regulation and genomic variability. DNA modifications alter the electrical signal as the DNA passes through the nanopore resulting in a differing picoampere signal for *k*-mers (*k* = 5 for R9; *k* = 9 for R10) containing modified bases compared to those consisting of only unmodified bases, see Fig. 10. Currently, 17 types of DNA modifications have been identified in the genomes of bacteria and eukaryotes [96], with the most well-characterized being methylation of cytosine (i.e. 5-methylcytosine, 5mC, and 5-hydroxymethylcytosine, 5hmC [97]) and adenine (i.e. N6-methyladenine, 6mA [98]).

Several computational tools have been developed to identify modifications from nanopore data, with a primary focus on 5mC and 6mA, using different computational approaches, in particular Hidden Markov Models, statistical tests, or neural networks, see Supplementary Table S2. The required input data for the methylation calling tools are usually the raw or basecalled fast5/pod5 files.

The DNA modification calling was traditionally performed after basecalling using tools like Nanopolish [99] or ONT’s Megalodon (https://github.com/nanoporetech/megalodon). Currently, ONT’s Dorado (https://github.com/nanoporetech/dorado) detects CpG methylations directly during basecalling if specified, and provides greater accuracy than tools like Nanopolish [100], which has difficulty when nearby CpG sites differ in methylation [99]. The performance of modification-detection tools varies by slight over-prediction (e.g. Nanopolish) or under-prediction (e.g. DeepMod) as described in several reviews [25, 100–103]. Validation of modifications remains essential, although tools like Nanopolish, Megalodon, DeepSignal, and Dorado already show high correlation with the gold standard whole-genome bisulfite sequencing (WGBS) [100, 101]. Meta approaches improve modification detection accuracy by combining the outputs of multiple tools. For example METEORE combines results from up to six tools using a random forest model, significantly improving accuracy but at the cost of increased runtime [25].

#### Detecting RNA modifications

The detection of RNA modifications using nanopore sequencing is less developed compared to DNA modifications, possibly because RNA is known to contain over 170 distinct modifications [104, 105]. RNA modifications are of the utmost importance for RNA function and regulation.

Computational tools exist only for detection of a handful of RNA modifications, see Supplementary Table S2. Aside from these downstream analysis tools, ONT’s Dorado directly enables modification detection by specifying specific basecalling models. Users can further customize the process by manually disabling additional filters as needed. Excitingly, basecallers like Bonito and Remora allow researchers to train custom models for detection of modifications of any kind. These models can be created from scratch or fine-tuned from pre-trained versions, offering flexibility for different datasets and research objectives. The models can perform binary classification to predict a certain modification, or directly call modified bases alongside the standard nucleotide sequence.

#### Detecting differential signals

Comparing raw ONT signals between different samples provides a powerful approach for detecting differential signals, which can point to mutations, modifications, and isotopic labels [106]. The goal of this approach is not necessarily to identify the exact type of modification but rather to detect the presence of a modification. The signal may differ in its measured pA range, including mean, variance, skewness, kurtosis, and dwell time. The calculation of these measures can be applied to full-length nanopore reads. To minimize normalization biases, comparative analyses should ideally be performed using the same flow cell. When comparisons across multiple flow cells are unavoidable, further normalization steps are needed.

On the other hand, for example, in the case of modifications such as deuterium in the DNA/RNA backbone [106], normalization can inadvertently obscure biologically meaningful information. This is particularly true when the signal-to-noise ratio—where the signal represents the modification and the noise stems from instrumental bias—is low. To address these challenges, carefully controlled experimental designs are essential to ensure that observed differences in the nanopore signal reflect genuine modifications rather than artifacts of the sequencing process.

## Conclusion

**Figure 11.**
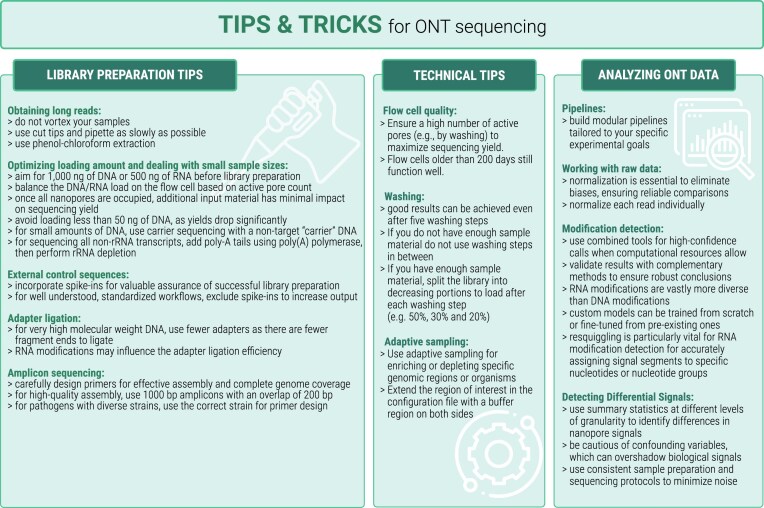
TIPS & TRICKS mentioned within the manuscript are summarized here for a better overview.

In summary, this study has yielded critical insights into refining nanopore sequencing using the MinION and GirdION sequencing platforms, with far-reaching implications for research and application. We displayed suggestions of nanopore sequencing for the user in the three categories: (i) library preparation tips; (ii) technical tips; and (iii) analyzing ONT data, see a summary in Fig. 11.

We reported general statistics for the number of sequenced bases after 12 h (EB12) on both R9 and R10 flow cells. As expected, the EB12 depended on the initial number of pores and the pore half time, which surprisingly varied depending on the sample type. In contrast, EB12 showed no correlation with the amount of input material, read length, or flow cell age. Additionally, we demonstrated that flow cells can still perform well after their expiration date. Meanwhile, we could also verify most of the observations on R10 flow cells. However, for the current study the sample size is too small for statistically robust conclusions.

With this publication, correlations and assertions from the ONT community have been statistically substantiated and are now citable for future work.

## Supplementary Material

gkag023_Supplemental_File

## Data Availability

No new data were generated or analyzed in support of this research.

## References

[1] Laszlo AH, Derrington IM, Ross BC et al. Decoding long nanopore sequencing reads of natural DNA. Nat Biotechnol. 2014;32:829–33. 10.1038/nbt.2950. 24964173 PMC4126851

[2] Wang Y, Zhao Y, Bollas A et al. Nanopore sequencing technology, bioinformatics and applications. Nat Biotechnol. 2021;39:1348–65. 10.1038/s41587-021-01108-x. 34750572 PMC8988251

[3] Petersen LM, Martin IW, Moschetti WE et al. Third-generation sequencing in the clinical laboratory: exploring the advantages and challenges of nanopore sequencing. J Clin Microbiol. 2019;58:e01315–19. 10.1128/JCM.01315-19. 31619531 PMC6935936

[4] Si HQ, Wang P, Long F et al. Cancer liquid biopsies by Oxford Nanopore Technologies sequencing of cell-free DNA: from basic research to clinical applications. Mol Cancer. 2024;23:265. 10.1186/s12943-024-02178-6. 39614371 PMC11605934

[5] Meyer D, Hennig A, Hums AB et al. Nanopore sequencing-derived methylation biomarker prediction for methylation-specific PCR in patients with head and neck squamous cell carcinoma. Clin Epigenetics. 2025;17:149. 10.1186/s13148-025-01960-7. 40946119 PMC12433006

[6] Nurk S, Koren S, Rhie A et al. The complete sequence of a human genome. Science. 2022;376:44–53. 10.1126/science.abj6987. 35357919 PMC9186530

[7] Nicholls SM, Quick JC, Tang S et al. Ultra-deep, long-read nanopore sequencing of mock microbial community standards. Gigascience. 2019;8:giz043. 10.1093/gigascience/giz043. 31089679 PMC6520541

[8] Overholt WA, Hölzer M, Geesink P et al. Inclusion of Oxford Nanopore long reads improves all microbial and viral metagenome-assembled genomes from a complex aquifer system. Environ Microbiol. 2020;22:4000–13. 10.1111/1462-2920.15186. 32761733

[9] Liu L, Yang Y, Deng Y et al. Nanopore long-read-only metagenomics enables complete and high-quality genome reconstruction from mock and complex metagenomes. Microbiome. 2022;10:209. 10.1186/s40168-022-01415-8. 36457010 PMC9716684

[10] Chen Z, Grim CJ, Ramachandran P et al. Advancing metagenome-assembled genome-based pathogen identification: unraveling the power of long-read assembly algorithms in Oxford Nanopore sequencing. Microbiol Spectr. 2024;12:e00117–24. 38687063 10.1128/spectrum.00117-24 PMC11237517

[11] Oikonomopoulos S, Bayega A, Fahiminiya S et al. Methodologies for transcript profiling using long-read technologies. Front Genet. 2020;11:606. 10.3389/fgene.2020.00606. 32733532 PMC7358353

[12] Jain M, Abu-Shumays R, Olsen HE et al. Advances in nanopore direct RNA sequencing. Nat Methods. 2022;19:1160–64. 10.1038/s41592-022-01633-w. 36203024 PMC11388133

[13] Rang FJ, Kloosterman WP, de Ridder J. From squiggle to basepair: computational approaches for improving nanopore sequencing read accuracy. Genome Biol. 2018;19:90. 10.1186/s13059-018-1462-9. 30005597 PMC6045860

[14] Garalde DR, Snell EA, Jachimowicz D et al. Highly parallel direct RNA sequencing on an array of nanopores. Nat Methods. 2018;15:201–6. 10.1038/nmeth.4577. 29334379

[15] Loose M, Rakyan V, Holmes N et al. Whale watching with BulkVis: a graphical viewer for Oxford Nanopore bulk fast5 files. Bioinformatics. 2019;35:2193–8. 30462145 10.1093/bioinformatics/bty841 PMC6596899

[16] Faria NR, Sabino EC, Nunes MR et al. Mobile real-time surveillance of Zika virus in Brazil. Genome Med. 2016;8:1–4. 10.1186/s13073-016-0356-2. 27683027 PMC5041528

[17] Amarasinghe SL, Su S, Dong X et al. Opportunities and challenges in long-read sequencing data analysis. Genome Biol. 2020;21:1–16. 10.1186/s13059-020-1935-5. PMC7006217 32033565

[18] Ip CLC, Loose M, Tyson JR et al. MinION Analysis and Reference Consortium: Phase 1 data release and analysis. F1000Res. 2015;4:1075. 10.12688/f1000research.7201.1. 26834992 PMC4722697

[19] Ferreira MR, Carratto TMT, Recalde TSF et al. Advances in forensic genetics: exploring the potential of long read sequencing. Forensic Sci Int Genet. 2024;74:103156. 39427416 10.1016/j.fsigen.2024.103156

[20] Sereika M, Kirkegaard RH, Karst SM et al. Oxford Nanopore R10. 4 long-read sequencing enables the generation of near-finished bacterial genomes from pure cultures and metagenomes without short-read or reference polishing. Nat Methods. 2022;19:823–6. 10.1038/s41592-022-01539-7. 35789207 PMC9262707

[21] Zhao W, Zeng W, Pang B et al. Oxford nanopore long-read sequencing enables the generation of complete bacterial and plasmid genomes without short-read sequencing. Front Microbiol. 2023;14:1179966. 10.3389/fmicb.2023.1179966. 37256057 PMC10225699

[22] Liu-Wei W, van der Toorn W, Bohn P et al. Sequencing accuracy and systematic errors of nanopore direct RNA sequencing. BMC Genomics. 2024;25:528. 10.1186/s12864-024-10440-w. 38807060 PMC11134706

[23] Magi A, Semeraro R, Mingrino A et al. Nanopore sequencing data analysis: state of the art, applications and challenges. Brief Bioinform. 2018;19:1256–72. 28637243 10.1093/bib/bbx062

[24] Leggett RM, Clark MD. A world of opportunities with nanopore sequencing. J Exp Bot. 2017;68:5419–29. 10.1093/jxb/erx289. 28992056

[25] Yuen ZWS, Srivastava A, Daniel R et al. Systematic benchmarking of tools for CpG methylation detection from nanopore sequencing. Nat Commun. 2021;12:3438. 10.1038/s41467-021-23778-6. 34103501 PMC8187371

[26] Martin S, Heavens D, Lan Y et al. Nanopore adaptive sampling: a tool for enrichment of low abundance species in metagenomic samples. Genome Biol. 2022;23:1–27. 10.1186/s13059-021-02582-x. 35067223 PMC8785595

[27] Timp W, Mirsaidov UM, Wang D et al. Nanopore sequencing: electrical measurements of the code of life. IEEE T Nanotechnol. 2010;9:281–94. 10.1109/TNANO.2010.2044418. PMC3092306 21572978

[28] Diederichs T, Pugh G, Dorey A et al. Synthetic protein-conductive membrane nanopores built with DNA. Nat Commun. 2019;10:5018. 10.1038/s41467-019-12639-y. 31685824 PMC6828756

[29] Howorka S, Siwy Z. Nanopore analytics: sensing of single molecules. Chem Soc Rev. 2009;38:2360–84. 10.1039/b813796j. 19623355

[30] Lindsay S . The promises and challenges of solid-state sequencing. Nat Nanotechnol. 2016;11:109–11. 10.1038/nnano.2016.9. 26839253 PMC5640258

[31] MacKenzie M, Argyropoulos C. An introduction to nanopore sequencing: past, present, and future considerations. Micromachines. 2023;14:459. 10.3390/mi14020459. 36838159 PMC9966803

[32] Jain M, Olsen HE, Paten B et al. The Oxford Nanopore MinION: delivery of nanopore sequencing to the genomics community. Genome Biol. 2016;17:1–11. 27887629 10.1186/s13059-016-1103-0 PMC5124260

[33] Logsdon GA, Vollger MR, Eichler EE. Long-read human genome sequencing and its applications. Nat Rev Genet. 2020;21:597–614. 10.1038/s41576-020-0236-x. 32504078 PMC7877196

[34] Sanderson ND, Kapel N, Rodger G et al. Comparison of R9. 4.1/Kit10 and R10/Kit12 Oxford Nanopore flowcells and chemistries in bacterial genome reconstruction. Microbial Genom. 2023;9:000910. 10.1099/mgen.0.000910 PMC9973852 36748454

[35] Ni Y, Liu X, Simeneh ZM et al. Benchmarking of Nanopore R10. 4 and R9. 4.1 flow cells in single-cell whole-genome amplification and whole-genome shotgun sequencing. Comput Struct Biotechnol J. 2023;21:2352–64. 10.1016/j.csbj.2023.03.038. 37025654 PMC10070092

[36] Castro-Wallace SL, Chiu CY, John KK et al. Nanopore DNA sequencing and genome assembly on the International Space Station. Sci Rep. 2017;7:18022. 10.1038/s41598-017-18364-0. 29269933 PMC5740133

[37] McIntyre AB, Rizzardi L, Yu AM et al. Nanopore sequencing in microgravity. NPJ Microgravity. 2016;2:1–9. 10.1038/npjmgrav.2016.35. 28725742 PMC5515536

[38] Kovaka S, Fan Y, Ni B et al. Targeted nanopore sequencing by real-time mapping of raw electrical signal with UNCALLED. Nat Biotechnol. 2021;39:431–41. 10.1038/s41587-020-0731-9. 33257863 PMC8567335

[39] Oxford Nanopore Technologies . MinKNOW: Mux scan and active channel selection. 2024. https://community.nanoporetech.com/nanopore_learning/lessons/mux-scan-and-acs [accessed: 21 December 2024].

[40] Koch C, Reilly-O’Donnell B, Gutierrez R et al. Nanopore sequencing of DNA-barcoded probes for highly multiplexed detection of microRNA, proteins and small biomarkers. Nat Nanotechnol. 2023;18:1483–91. 10.1038/s41565-023-01479-z. 37749222 PMC10716039

[41] Prall TM, Neumann EK, Karl JA et al. Consistent ultra-long DNA sequencing with automated slow pipetting. BMC genomics. 2021;22:1–12. 10.1186/s12864-021-07500-w. 33711930 PMC7953553

[42] ‘Giron’Koetsier PA, Cantor EJ. A simple approach for effective shearing and reliable concentration measurement of ultra-high-molecular-weight DNA. BioTechniques. 2021;71:439–44. 10.2144/btn-2021-0051. 34232102

[43] Jones A, Torkel C, Stanley D et al. High-molecular weight DNA extraction, clean-up and size selection for long-read sequencing. PLoS One. 2021;16:e0253830. 10.1371/journal.pone.0253830. 34264958 PMC8282028

[44] Maghini DG, Moss EL, Vance SE et al. Improved high-molecular-weight DNA extraction, nanopore sequencing and metagenomic assembly from the human gut microbiome. Nat Protoc. 2021;16:458–71. 10.1038/s41596-020-00424-x. 33277629 PMC8750633

[45] Zhang Y, Zhang Y, Burke JM et al. A simple thermoplastic substrate containing hierarchical silica lamellae for high-molecular-weight DNA extraction. Adv Mater. 2016;28:10630–6. 10.1002/adma.201603738. 27862402 PMC5234087

[46] Jaudou S, Tran ML, Vorimore F et al. Evaluation of high molecular weight DNA extraction methods for long-read sequencing of Shiga toxin-producing Escherichia coli. PLoS One. 2022;17:e0270751. 10.1371/journal.pone.0270751. 35830426 PMC9278759

[47] Sultan M, Amstislavskiy V, Risch T et al. Influence of RNA extraction methods and library selection schemes on RNA-seq data. BMC Genomics. 2014;15:1–13. 10.1186/1471-2164-15-675. 25113896 PMC4148917

[48] Wood DE, Lu J, Langmead B. Improved metagenomic analysis with Kraken 2. Genome Biol. 2019;20:1–13. 10.1186/s13059-019-1891-0. 31779668 PMC6883579

[49] Browne PD, Nielsen TK, Kot W et al. GC bias affects genomic and metagenomic reconstructions, underrepresenting GC-poor organisms. Gigascience. 2020;9:giaa008. 10.1093/gigascience/giaa008. 32052832 PMC7016772

[50] Delahaye C, Nicolas J. Sequencing DNA with nanopores: Troubles and biases. PLoS One. 2021;16:e0257521. 10.1371/journal.pone.0257521. 34597327 PMC8486125

[51] Heavens D, Chooneea D, Giolai M et al. How low can you go? Driving down the DNA input requirements for nanopore sequencing. bioRxiv, 10.1101/2021.10.15.464554, 18 October 2021, preprint: not peer reviewed.

[52] Mojarro A, Hachey J, Ruvkun G et al. CarrierSeq: a sequence analysis workflow for low-input nanopore sequencing. BMC Bioinformatics. 2018;19:108. 29587645 10.1186/s12859-018-2124-3 PMC5872496

[53] Arakawa K . Ultralow-input genome library preparation for nanopore sequencing with droplet MDA. Methods Mol Biol. 2023;2632:91–100. 36781723 10.1007/978-1-0716-2996-3_7

[54] Wang Q, Bönigk S, Böhm V et al. Single-cell transcriptome sequencing on the Nanopore platform with ScNapBar. RNA. 2021;27:763–70. 10.1261/rna.078154.120. 33906975 PMC8208055

[55] Wang C, Qiu J, Liu M et al. Microfluidic biochips for single-cell isolation and single-cell analysis of multiomics and exosomes. Adv Sci (Weinh). 2024;11:e2401263. 10.1002/advs.202401263. 38767182 PMC11267386

[56] Simon SA, Schmidt K, Griesdorn L et al. Dancing the Nanopore limbo—nanopore metagenomics from small DNA quantities for bacterial genome reconstruction. BMC Genomics. 2023;24:727. 38041056 10.1186/s12864-023-09853-w PMC10693096

[57] Scott M, Gunderson CW, Mateescu EM et al. Interdependence of cell growth and gene expression: origins and consequences. Science. 2010;330:1099–102. 21097934 10.1126/science.1192588

[58] Marzluff WF, Wagner EJ, Duronio RJ. Metabolism and regulation of canonical histone mRNAs: life without a poly(A) tail. Nat Rev Genet. 2008;9:843–54. 18927579 10.1038/nrg2438 PMC2715827

[59] Turowski TW, Boguta M. Specific features of RNA polymerases I and III: structure and assembly. Front Mol Biosci. 2021;8:680090. 34055890 10.3389/fmolb.2021.680090 PMC8160253

[60] O’Neil D, Glowatz H, Schlumpberger M. Ribosomal RNA depletion for efficient use of RNA-seq capacity. Curr Protoc Mol Biol. 2013;Chapter 4:Unit 4.19. 10.1002/0471142727.mb0419s103 23821444

[61] Fang Y, Changavi A, Yang M et al. Nanopore whole transcriptome analysis and pathogen surveillance by a novel solid-phase catalysis approach. Adv Sci (Weinh). 2022;9:e2103373. 10.1002/advs.202103373. 34837482 PMC8787394

[62] Lundberg DS, Yourstone S, Mieczkowski P et al. Practical innovations for high-throughput amplicon sequencing. Nat Methods. 2013;10:999–1002. 10.1038/nmeth.2634. 23995388

[63] Kilianski A, Haas JL, Corriveau EJ et al. Bacterial and viral identification and differentiation by amplicon sequencing on the MinION nanopore sequencer. Gigascience. 2015;4:s13742–015. 10.1186/s13742-015-0051-z. PMC4374364 25815165

[64] Lee GY, Park K, Lee YS et al. Molecular diagnosis of patients with hepatitis A virus infection using amplicon-based nanopore sequencing. PLoS One. 2023;18:e0288361. 10.1371/journal.pone.0288361. 37437048 PMC10337952

[65] Butt SL, Taylor TL, Volkening JD et al. Rapid virulence prediction and identification of Newcastle disease virus genotypes using third-generation sequencing. Virol J. 2018;15:179. 10.1186/s12985-018-1077-5. 30466441 PMC6251111

[66] Butt SL, Erwood EC, Zhang J et al. Real-time, MinION-based, amplicon sequencing for lineage typing of infectious bronchitis virus from upper respiratory samples. J Vet Diagn Invest. 2020;33:179–90. 10.1177/1040638720910107. 32133932 PMC7201198

[67] Grubaugh ND, Gangavarapu K, Quick J et al. An amplicon-based sequencing framework for accurately measuring intrahost virus diversity using PrimalSeq and iVar. Genome Biol. 2019;20:8. 10.1186/s13059-018-1618-7. 30621750 PMC6325816

[68] Freed NE, Vlková M, Faisal MB et al. Rapid and inexpensive whole-genome sequencing of SARS-CoV-2 using 1200 bp tiled amplicons and Oxford Nanopore Rapid Barcoding. Biol Methods Protoc. 2020;5:bpaa014. 10.1093/biomethods/bpaa014. 33029559 PMC7454405

[69] Whitford W, Hawkins V, Moodley KS et al. Proof of concept for multiplex amplicon sequencing for mutation identification using the MinION nanopore sequencer. Sci Rep. 2022;12:8572. 10.1038/s41598-022-12613-7. 35595858 PMC9122479

[70] McCrone JT, Woods RJ, Martin ET et al. Stochastic processes constrain the within and between host evolution of influenza virus. Elife. 2018;7:e35962. 10.7554/eLife.35962. 29683424 PMC5933925

[71] Gilbert KB, Fahlgren N, Kasschau KD et al. Preparation of multiplexed small RNA libraries from plants. Bio Protoc. 2014;4:e1275. 10.21769/BioProtoc.1275. PMC4675356 26661568

[72] Taylor BC, Lejzerowicz F, Poirel M et al. Consumption of fermented foods is associated with systematic differences in the gut microbiome and metabolome. mSystems. 2020;5:e00901–19. 32184365 10.1128/mSystems.00901-19 PMC7380580

[73] Brandt C, Krautwurst S, Spott R et al. PoreCov-an easy to use, fast, and robust workflow for SARS-CoV-2 genome reconstruction via nanopore sequencing. Front Genet. 2021;12:711437. 10.3389/fgene.2021.711437. 34394197 PMC8355734

[74] Oxford Nanopore Technologies plc . Direct RNA Sequencing (SQK-RNA004) Protocol [Technical protocol]. 2025. Accessed: November 2025.

[75] AbouHaidar MG, Ivanov IG. Non-enzymatic RNA hydrolysis promoted by the combined catalytic activity of buffers and magnesium ions. Z Naturforsch C J Biosci. 1999;54:542–48. 10.1515/znc-1999-7-813. 10488562

[76] Kornienko IV, Aramova OY, Tishchenko AA et al. RNA stability: a review of the role of structural features and environmental conditions. Molecules. 2024;29:5978. 10.3390/molecules29245978. 39770066 PMC11676819

[77] Champoux JJ, Schultz SJ. Ribonuclease H: properties, substrate specificity and roles in retroviral reverse transcription. FEBS J. 2009;276:1506–16. 10.1111/j.1742-4658.2009.06909.x. 19228195 PMC2742777

[78] Tian L, Kim MS, Li H et al. Structure of HIV-1 reverse transcriptase cleaving RNA in an RNA/DNA hybrid. Proc Natl Acad Sci. 2018;115:507–12. 10.1073/pnas.1719746115. 29295939 PMC5777007

[79] Payne A, Holmes N, Clarke T et al. Readfish enables targeted nanopore sequencing of gigabase-sized genomes. Nat Biotechnol. 2021;39:442–50. 10.1038/s41587-020-00746-x. 33257864 PMC7610616

[80] Filser M, Schwartz M, Merchadou K et al. Adaptive nanopore sequencing to determine pathogenicity of BRCA1 exonic duplication. J Med Genet. 2023;60:1206–9. 10.1136/jmg-2023-109155. 37263769 PMC10715497

[81] De Meulenaere K, Cuypers WL, Gauglitz JM et al. Selective whole-genome sequencing of Plasmodium parasites directly from blood samples by Nanopore adaptive sampling. Mbio. 2024;15:e01967–23. 10.1128/mbio.01967-23. 38054750 PMC10790762

[82] Marquet M, Zöllkau J, Pastuschek J et al. Evaluation of microbiome enrichment and host DNA depletion in human vaginal samples using Oxford Nanopore’s adaptive sequencing. Sci Rep. 2022;12:4000. 10.1038/s41598-022-08003-8. 35256725 PMC8901746

[83] Conesa A, Madrigal P, Tarazona S et al. A survey of best practices for RNA-seq data analysis. Genome Biol. 2016;17:13. 10.1186/s13059-016-0881-8. 26813401 PMC4728800

[84] Leger A, Leonardi T. pycoQC, interactive quality control for Oxford Nanopore Sequencing. J Open Source Softw. 2019;4:1236. 10.21105/joss.01236.

[85] De Coster W, Rademakers R. NanoPack2: population-scale evaluation of long-read sequencing data. Bioinformatics. 2023;39:btad311. 10.1093/bioinformatics/btad311. 37171891 PMC10196664

[86] Li H . Minimap2: pairwise alignment for nucleotide sequences. Bioinformatics. 2018;34:3094–100. 10.1093/bioinformatics/bty191. 29750242 PMC6137996

[87] Jain C, Rhie A, Zhang H et al. Weighted minimizer sampling improves long read mapping. Bioinformatics. 2020;36:i111–8. 10.1093/bioinformatics/btaa435. 32657365 PMC7355284

[88] Boostrom I, Portal EAR, Spiller OB et al. Comparing long-read assemblers to explore the potential of a sustainable low-cost, low-infrastructure approach to sequence antimicrobial resistant bacteria with Oxford Nanopore Sequencing. Front Microbiol. 2022;13:796465. 10.3389/fmicb.2022.796465. 35308384 PMC8928191

[89] Shafin K, Pesout T, Lorig-Roach R et al. Nanopore sequencing and the Shasta toolkit enable efficient de novo assembly of eleven human genomes. Nat Biotechnol. 2020;38:1044–53. 10.1038/s41587-020-0503-6. 32686750 PMC7483855

[90] Hu J, Wang Z, Sun Z et al. NextDenovo: an efficient error correction and accurate assembly tool for noisy long reads. Genome Biol. 2024;25:107. 38671502 10.1186/s13059-024-03252-4 PMC11046930

[91] Sun J, Li R, Chen C et al. Benchmarking Oxford Nanopore read assemblers for high-quality molluscan genomes. Philos Trans R Soc Lond B Biol Sci. 2021;376:20200160. 10.1098/rstb.2020.0160. 33813888 PMC8059532

[92] Vaser R, Sović I, Nagarajan N et al. Fast and accurate de novo genome assembly from long uncorrected reads. Genome Res. 2017;27:737–46. 10.1101/gr.214270.116. 28100585 PMC5411768

[93] Gholami A, Kim S, Dong Z et al. A survey of quantization methods for efficient neural network inference. Low-power computer vision. 2022;291–326.

[94] Fleming AM, Mathewson NJ, Manage SAH et al. Nanopore dwell time analysis permits sequencing and conformational assignment of *Pseudouridine* in SARS-CoV-2. ACS Cent Sci. 2021;7:1707–17. 10.1021/acscentsci.1c00788. 34729414 PMC8554835

[95] Gamaarachchi H, Lam CW, Jayatilaka G et al. GPU accelerated adaptive banded event alignment for rapid comparative nanopore signal analysis. BMC Bioinformatics. 2020;21:1–13. 10.1186/s12859-020-03697-x. 32758139 PMC7430849

[96] Raiber EA, Hardisty R, van Delft P et al. Mapping and elucidating the function of modified bases in DNA. Nat Rev Chem. 2017;1:0069. 10.1038/s41570-017-0069.

[97] Klungland A, Robertson AB. Oxidized C5-methyl cytosine bases in DNA: 5-hydroxymethylcytosine; 5-formylcytosine; and 5-carboxycytosine. Free Radical Bio Med. 2017;107:62–8. 10.1016/j.freeradbiomed.2016.11.038. 27890639

[98] Heyn H, Esteller M. An adenine code for DNA: a second life for N6-methyladenine. Cell. 2015;161:710–3. 10.1016/j.cell.2015.04.021. 25936836

[99] Simpson JT, Workman RE, Zuzarte P et al. Detecting DNA cytosine methylation using nanopore sequencing. Nat Methods. 2017;14:407–10. 10.1038/nmeth.4184. 28218898

[100] Sigurpalsdottir BD, Stefansson OA, Holley G et al. A comparison of methods for detecting DNA methylation from long-read sequencing of human genomes. Genome Biol. 2024;25:69. 10.1186/s13059-024-03207-9. 38468278 PMC10929077

[101] Liu Y, Rosikiewicz W, Pan Z et al. DNA methylation-calling tools for Oxford Nanopore sequencing: a survey and human epigenome-wide evaluation. Genome Biol. 2021;22:1–33. 10.1186/s13059-021-02510-z. 34663425 PMC8524990

[102] Yao B, Hsu C, Goldner G et al. Nanopore callers for epigenetics from limited supervised data. bioRxiv, 10.1101/2021.06.17.448800, 17 June 2021, preprint: not peer reviewed.

[103] Akbari V, Garant JM, O’Neill K et al. Megabase-scale methylation phasing using nanopore long reads and NanoMethPhase. Genome Biol. 2021;22:1–21. 10.1186/s13059-021-02283-5. 33618748 PMC7898412

[104] Chen H, Yao J, Bao R et al. Cross-talk of four types of RNA modification writers defines tumor microenvironment and pharmacogenomic landscape in colorectal cancer. Mol Cancer. 2021;20:29. 10.1186/s12943-021-01322-w. 33557837 PMC7869236

[105] Cappannini A, Ray A, Purta E et al. MODOMICS: a database of RNA modifications and related information. 2023 update. Nucleic Acids Research. 2023;52:D239–44. 10.1093/nar/gkad1083. PMC10767930 38015436

[106] Höner zu Siederdissen C, Spangenberg J, Bisdorf K et al. Nanopore sequencing enables novel detection of deuterium incorporation in DNA. Comput Struct Biotechnol J. 2024;23:3584–94. 10.1016/j.csbj.2024.09.027. 39963424 PMC11832021

