# Supplementary material for: Unlocking the full potential of nanopore sequencing: tips, tricks, and advanced data analysis techniques
Source: Nucleic Acids Res. 2026 Feb 2;54(3):gkag023. doi: 10.1093/nar/gkag023 (PMC12862388; doi:10.1093/nar/gkag023)
Supplement: gkag023_Supplemental_File [file gkag023_supplemental_file.pdf]

SUPPLEMENTARY INFORMATION

Table S1: **Sample Summary** – overview of all runs used for the analysis of Fig. 2, Fig. 4 and Fig. S4 and Fig. S5–S8. ID – unique identifier, with post-decimal number indicating wash count and citation if externally sequencing; RNA/DNA – RNA or DNA sequencing; fc version – R9 or R10 flow cells; device – MinION or GridION device; #cha – amount of active channels at the begin of the sequencing run; #por – amount of active pores at the beginning of the sequencing run; fc age – time passed since delivery of the Flow cell in days; sample type – from which kind of species the sequenced material originated in nine categories: virus, bacteria, protist, metagenomic, insect, mouse, human; buffer – LFB, SFB or RNA buffer; ng load – amount of loaded library (DNA/RNA) in ng; estimated bases – amount of bases sequenced in mega bases (mb), estimated by sequencing software MinKNOW; EB 12 – amount of sequenced bases after 12 h in mega bases (mb), estimated by sequencing software MinKNOW; mean rl – mean estimated read length of all sequenced reads in bases; pore ht – calculated pore half time of the flow cell in hours (see main text); library prep – protocol used during library preparation

ID (citation)	RNA/ DNA	fc version	device	#cha	#por	fc age	sample type	buffer	ng load	estimated bases (Mb)	EB12 (Mb)	mean rl	pore ht	fc id	library prep
1.1 (107)	DNA	R10	GridIon	510	NA	NA	virus	SFB	NA	8161	4721	2036	998	FAU91805	NBD114-24
2.1 (107)	DNA	R10	GridIon	480	NA	NA	virus	SFB	NA	4745	4745	2934	1630	FAX97604	NBD114-24
3.1 (107)	DNA	R10	GridIon	467	1766	NA	virus	SFB	NA	6041	3977	2137	643	FAY00760	NBD114-24
4.1 (107)	DNA	R10	GridIon	502	1860	NA	virus	SFB	NA	8139	5074	2640	1012	FAY01081	NBD114-96
5.1 (107)	DNA	R10	GridIon	501	1869	NA	virus	SFB	NA	9339	5444	3776	1397	FAY01249	NBD114-96
6.1 (107)	DNA	R10	GridIon	504	NA	NA	virus	SFB	NA	9932	5699	4022	1510	FAY01385	NBD114-96
7.1	DNA	R10	MinIon	293	1368	172	virus	SFB	60	177	153	419	51	FAO88286	LSK-109
8.1	RNA	R9	MinIon	295	749	174	virus	RNA	140	523	423	1530	900	FAM95379	RNA002
9.1	RNA	R9	MinIon	487	1333	56	virus	RNA	210	1571	923	1700	NA	FAO07649	RNA002
10.2	RNA	R9	MinIon	489	1385	49	virus	RNA	90	1495	1031	1454	NA	FAO10418	RNA002
11.1	RNA	R9	MinIon	501	1282	10	virus	RNA	74	1324	591	1072	1638	FAO53185	RNA002
12.1	RNA	R9	MinIon	506	1372	112	virus	RNA	308	1247	885	1046	797	FAO83518	RNA002
13.1	RNA	R9	MinIon	473	1201	127	virus	RNA	186	1326	869	1577	810	FAO86549	RNA002
14.1	RNA	R9	MinIon	470	1201	102	virus	RNA	304	118	117	1206	79	FAO86555	RNA002
15.1	RNA	R9	MinIon	505	1380	111	virus	RNA	392	1028	801	1123	637	FAO86826	RNA002
16.1	RNA	R9	MinIon	510	1342	34	virus	RNA	256	483	462	982	269	FAO86858	RNA002
17.1	RNA	R9	MinIon	500	1241	111	virus	RNA	348	136	136	1112	113	FAO86895	RNA002
18.1	RNA	R9	MinIon	500	1308	110	virus	RNA	266	626	580	1099	414	FAO86907	RNA002
19.1	RNA	R9	MinIon	498	1298	110	virus	RNA	420	296	296	1140	212	FAO86991	RNA002
20.1	RNA	R9	MinIon	442	1142	93	virus	RNA	224	284	283	1354	225	FAO86996	RNA002
21.1	RNA	R9	MinIon	495	1228	111	virus	RNA	360	314	312	1133	215	FAO87031	RNA002
22.1	RNA	R9	MinIon	482	1249	127	virus	RNA	276	295	294	1353	232	FAO87106	RNA002
23.1	RNA	R9	MinIon	432	1002	9	virus	RNA	167	1400	650	1677	1348	FAO88388	RNA002
24.1	DNA	R9	MinIon	512	1960	3	virus	SFB	575	13251	6708	765	1439	FAO92019	LSK109
25.1	RNA	R9	MinIon	491	1327	110	virus	RNA	346	600	556	1111	352	FAO92042	RNA002
26.1	DNA	R9	MinIon	511	1907	6	virus	SFB	516	9748	6396	866	958	FAO92112	LSK109
27.1	RNA	R9	MinIon	497	1307	110	virus	RNA	356	768	657	1123	526	FAO92116	RNA002
28.1	RNA	R9	MinIon	502	1365	111	virus	RNA	254	1141	847	975	795	FAO92254	RNA002
29.1	RNA	R9	MinIon	322	893	93	virus	RNA	236	317	316	1151	368	FAO92303	RNA002
30.1	RNA	R9	MinIon	350	1210	167	virus	RNA	268	1708	767	1666	1373	FAP02359	RNA002
31.1	RNA	R9	MinIon	93	152	51	virus	RNA	580	46	44	1256	166	FAQ01609	RNA002
31.2	RNA	R9	MinIon	NA	814	NA	virus	RNA	580	NA	487	1246	557	FAQ01609	RNA002
32.1	RNA	R9	MinIon	420	965	147	virus	RNA	52	7	7	605	53	FAQ69187	RNA002
33.1 (108)	DNA	R10	GridIon	510	1609	NA	bacteria	LFB	NA	22320	4567	10233	3562	FAU91295	NBD114-24
34.1 (108)	DNA	R10	GridIon	511	1556	NA	bacteria	LFB	NA	10173	3988	7108	2071	FAU92937	NBD114-24
35.1 (108)	DNA	R10	GridIon	502	1491	NA	bacteria	LFB	NA	12166	3944	7911	1930	FAW21704	NBD114-24
36.1 (108)	DNA	R10	GridIon	510	1460	NA	bacteria	LFB	NA	12767	3779	1553	2366	FAW22654	NBD114-24
37.1 (108)	DNA	R10	GridIon	510	1578	NA	bacteria	LFB	NA	14943	4433	3423	2026	FAW22671	NBD114-24
38.1 (108)	DNA	R10	GridIon	348	1019	NA	bacteria	LFB	NA	6458	2713	1629	1675	FAW23628	NBD114-24
39.1 (108)	DNA	R10	GridIon	498	1567	NA	bacteria	LFB	NA	6065	3604	6144	719	FAW45145	NBD114-24
40.1 (108)	DNA	R10	GridIon	510	1566	NA	bacteria	LFB	NA	14424	4395	8707	2285	FAW45147	NBD114-24
41.1 (108)	DNA	R10	GridIon	478	1501	NA	bacteria	LFB	NA	7288	3437	9545	1050	FAW45167	NBD114-24
42.1 (108)	DNA	R10	GridIon	413	1262	NA	bacteria	LFB	NA	2715	2177	1855	456	FAW98004	NBD114-24
43.1 (108)	DNA	R10	GridIon	503	NA	NA	bacteria	LFB	NA	3563	NA	NA	NA	FAW98056	NBD114-24
44.1 (108)	DNA	R10	GridIon	499	1899	NA	bacteria	LFB	NA	13610	6244	3383	1169	FAW99941	NBD114-24
45.1 (108)	DNA	R10	GridIon	495	NA	NA	bacteria	LFB	NA	4855	NA	NA	NA	FAX00084	NBD114-24
46.1 (108)	DNA	R10	GridIon	484	1484	NA	bacteria	LFB	NA	7281	3929	2441	851	FAX00224	NBD114-24
47.1	DNA	R10	GridIon	487	1812	105	bacteria	LFB	1216	11256	4949	3599	934	FAX12900	NBD114-24
48.1	DNA	R10	GridIon	221	813	112	bacteria	LFB	403	4209	2002	3186	657	FAX13587	NBD114-24
49.1	DNA	R10	GridIon	511	NA	98	bacteria	LFB	552	14912	5538	1931	NA	FAX14134	NBD114-24
50.1	DNA	R10	GridIon	506	NA	105	bacteria	LFB	324	10673	4891	2555	NA	FAX18203	NBD114-24
51.1 (108)	DNA	R10	GridIon	484	1844	NA	bacteria	LFB	NA	21675	5881	3121	2151	FAX70780	NBD114-24
52.1	DNA	R10	MinIon	235	999	186	bacteria	SFB	139.2	1239	770	1169	1531	FAO85473	LSK109
53.1 (109)	DNA	R10	MinIon	348	NA	36	bacteria	LFB	NA	2625	NA	NA	NA	FAS37108	NBD112-24
53.2 (109)	DNA	R10	MinIon	428	1116	35	bacteria	LFB	1008	1242	1041	6008	NA	FAS37108	NBD112-24
54.1 (109)	DNA	R10	MinIon	132	NA	31	bacteria	LFB	298	997	707	9448	NA	FAS38416	NBD112-24
54.2 (109)	DNA	R10	MinIon	417	NA	30	bacteria	LFB	276	4825	3281	9328	NA	FAS38416	NBD112-24
55.1 (109)	DNA	R10	MinIon	229	NA	1	bacteria	LFB	1440	651	443	2796	NA	FAV74660	NBD112-24
56.1 (110)	DNA	R10	MinIon	500	NA	7	bacteria	LFB	157	11585	4816	3574	NA	FAW66139	NBD114-24
57.1 (110)	DNA	R10	MinIon	468	NA	2	bacteria	LFB	NA	9767	4480	5356	NA	FAW73005	NBD114-24
57.2 (110)	DNA	R10	MinIon	NA	NA	1	bacteria	LFB	NA	NA	NA	NA	NA	FAW73005	NBD114-24
58.1 (110)	DNA	R10	MinIon	226	NA	1	bacteria	LFB	199	4678	NA	NA	NA	FAW75095	NBD114-24

ID (citation)	RNA/ DNA	fc version	device	#cha	#por	fc age	sample type	buffer	ng load	estimated bases (Mb)	EB12 (Mb)	mean rl	pore ht	fc id	library prep
59.1 (109)	DNA	R10	MinIon	487	NA	1	bacteria	LFB	226	0	NA	NA	NA	FAX18284	NBD114-24
60.1 (110)	DNA	R10	MinIon	473	NA	1	bacteria	LFB	154	12573	4714	4304	NA	FAX30985	NBD114-24
61.1 (108)	DNA	R9	GridIon	484	1447	NA	bacteria	LFB	NA	9421	3623	7487	1791	FAU91863	LSK114
62.1 (108)	DNA	R9	GridIon	511	1398	NA	bacteria	LFB	NA	6066	3190	13070	2225	FAV24719	LSK109
63.1 (108)	DNA	R9	GridIon	494	1512	NA	bacteria	LFB	NA	18854	6177	9601	2187	FAV30532	LSK109
64.1	DNA	R9	MinIon	139	482	101	bacteria	LFB	200	100	NA	5093	18	FAO16410	LSK109
65.1	DNA	R9	MinIon	499	1439	4	bacteria	SFB	238	18063	6136	6785	2038	FAO53260	LSK109
66.1	DNA	R9	MinIon	416	1021	114	bacteria	SFB	352	11310	4237	3302	2384	FAO87074	LSK109
67.4	DNA	R9	MinIon	449	1541	37	bacteria	LFB	200	654	NA	4932	NA	FAO92001	LSK109
68.1	DNA	R9	MinIon	416	1161	178	bacteria	SFB	600	7394	4172	2023	1108	FAP00827	LSK109
69.1	DNA	R9	MinIon	449	1659	121	bacteria	SFB	775	2507	NA	18442	NA	FAQ01004	LSK109
69.2	DNA	R9	MinIon	355	1393	122	bacteria	SFB	444	5911	3731	12254	2	FAQ01004	LSK109
70.1	DNA	R9	MinIon	494	1620	44	bacteria	SFB	1428	8812	4452	1262	1652	FAQ58659	LSK109
71.1	DNA	R9	MinIon	454	1692	58	bacteria	SFB	504	18406	5950	20443	2303	FAQ58677	LSK109
72.1	DNA	R9	MinIon	493	1312	41	bacteria	SFB	479.6	25403	6853	2023	2787	FAQ69194	LSK109
73.1	DNA	R9	MinIon	480	1468	87	bacteria	LFB	700	23019	6807	23746	2554	FAR96927	LSK109
74.1	DNA	R9	MinIon	406	1398	14	protist	LFB	600	4265	3028	1592	446	FAQ01569	LSK109
75.1	DNA	R9	MinIon	485	1692	80	protist	SFB	310	4620	3452	1988	608	FAQ01665	LSK109
76.1	DNA	R9	MinIon	508	1881	16	protist	SFB	528	10925	5862	1852	1169	FAQ01773	LSK109
77.1	DNA	R9	MinIon	499	1785	78	protist	SFB	470	4948	3880	1867	557	FAQ01847	LSK109
77.2	DNA	R9	MinIon	371	1329	79	protist	SFB	160	2085	1749	2093	258	FAQ01847	LSK109
78.1	DNA	R10	GridIon	503	1719	NA	metagenomic	LFB	NA	5046	2434	5637	821	FAW10206	RBK114-24
79.1	DNA	R10	GridIon	501	1723	NA	metagenomic	LFB	NA	5681	2805	5293	932	FAW13592	RBK114-24
80.1	DNA	R10	GridIon	457	1556	NA	metagenomic	LFB	NA	5468	2379	6421	1184	FAX33641	RBK114-24
81.1	DNA	R10	GridIon	500	1733	NA	metagenomic	LFB	NA	7313	3103	5179	1068	FAX57960	RBK114-24
82.1	DNA	R10	GridIon	507	1657	NA	metagenomic	LFB	NA	5308	2229	7428	1191	FAX59252	RBK114-24
83.1	DNA	R10	MinIon	450	1657	156	metagenomic	SFB	600	7086	3361	1648	1199	FAO79888	LSK109
84.1	DNA	R9	MinIon	NA	1953	NA	metagenomic	SFB	447	NA	7190	1355	2371	FAK75664	LSK109
85.1	DNA	R9	MinIon	NA	1921	NA	metagenomic	SFB	471.6	NA	6634	1246	2878	FAL78178	LSK109
85.2	DNA	R9	MinIon	NA	19	NA	metagenomic	SFB	471.6	NA	NA	836	NA	FAL78178	LSK109
86.1	DNA	R9	MinIon	NA	186	NA	metagenomic	SFB	280	NA	555	2355	NA	FAL78306	LSK109
86.2	DNA	R9	MinIon	NA	1915	NA	metagenomic	SFB	280	NA	7182	3067	3253	FAL78306	LSK109
87.1	DNA	R9	MinIon	507	1933	154	metagenomic	SFB	252	17853	7006	2561	2339	FAL80840	LSK109
87.2	DNA	R9	MinIon	396	1452	155	metagenomic	SFB	252	10310	5187	2378	1001	FAL80840	LSK109
88.1	DNA	R9	MinIon	512	NA	167	metagenomic	SFB	256	19674	7000	2717	3098	FAL81216	LSK109
88.2	DNA	R9	MinIon	378	1279	168	metagenomic	SFB	256	9050	4769	2434	1110	FAL81216	LSK109
89.1	DNA	R9	MinIon	506	1793	147	metagenomic	SFB	301	6030	4453	1833	729	FAL83332	LSK109
89.2	DNA	R9	MinIon	457	1617	148	metagenomic	SFB	301	2492	2204	1124	271	FAL83332	LSK109
90.1	DNA	R9	MinIon	510	1966	179	metagenomic	SFB	1300	27326	6719	2119	2953	FAL83351	LSK109
91.1	DNA	R9	MinIon	479	1813	177	metagenomic	SFB	431	13234	6063	1166	1671	FAL84179	LSK109
92.1	DNA	R9	MinIon	485	1802	171	metagenomic	SFB	379	19643	5541	2215	2231	FAL85078	LSK109
93.1	DNA	R9	MinIon	508	NA	150	metagenomic	SFB	360	14580	7302	4228	2041	FAL91762	LSK109
93.2	DNA	R9	MinIon	420	1588	151	metagenomic	SFB	360	13564	5661	2288	1945	FAL91762	LSK109
94.1	DNA	R9	MinIon	507	1891	181	metagenomic	SFB	400	17020	5792	1623	2302	FAM93261	LSK109
95.1	DNA	R9	MinIon	376	1365	174	metagenomic	SFB	410	8949	4597	2509	1247	FAM95301	LSK109
96.1	DNA	R9	MinIon	503	1843	190	metagenomic	SFB	609	25293	6351	1795	2686	FAM95308	LSK109
97.1	DNA	R9	MinIon	508	1945	181	metagenomic	SFB	458	26777	7184	3221	2585	FAM95383	LSK109
98.1	DNA	R9	MinIon	271	1005	174	metagenomic	SFB	300	2850	2272	2305	606	FAM96150	LSK109
99.1	DNA	R9	MinIon	503	1896	178	metagenomic	SFB	564	17602	5838	1871	2520	FAM96240	LSK109
100.1	DNA	R9	MinIon	465	1691	171	metagenomic	SFB	253	8206	5247	3313	1363	FAM96327	LSK109
100.2	DNA	R9	MinIon	353	1293	172	metagenomic	SFB	253	6930	4180	1905	1291	FAM96327	LSK109
101.1	DNA	R9	MinIon	507	1876	190	metagenomic	SFB	552	28264	6597	1462	3230	FAM96767	LSK109
102.1	DNA	R9	MinIon	481	1780	54	metagenomic	SFB	460	12460	5585	1483	1076	FAO10159	LSK109
103.1	DNA	R9	MinIon	499	1771	39	metagenomic	SFB	372	2181	1580	1713	812	FAO10214	LSK109
103.2	DNA	R9	MinIon	240	796	45	metagenomic	SFB	460	3263	2633	1585	850	FAO10214	LSK109
104.1	DNA	R9	MinIon	507	1594	40	metagenomic	SFB	960	4117	2129	922	1157	FAO10253	LSK109
105.1	DNA	R9	MinIon	491	1602	41	metagenomic	SFB	408	2037	1597	1785	NA	FAO10336	LSK109
106.1	DNA	R9	MinIon	498	1742	39	metagenomic	SFB	393	4078	2218	1444	1008	FAO10413	LSK109
10.5	DNA	R9	MinIon	337	1157	55	metagenomic	SFB	564	3713	3571	1340	644	FAO10418	LSK109
107.1	DNA	R9	MinIon	506	1948	42	metagenomic	SFB	634	23235	7055	1672	2661	FAO12115	LSK109
108.1	DNA	R9	MinIon	510	1955	42	metagenomic	SFB	538	18522	6564	1740	2241	FAO12140	LSK109
109.1	DNA	R9	MinIon	501	1881	42	metagenomic	SFB	600	18284	6171	1912	2292	FAO12169	LSK109
110.1	DNA	R9	MinIon	484	NA	20	metagenomic	SFB	672	10957	5227	2273	1750	FAO16442	LSK109
111.1	DNA	R9	MinIon	512	1853	48	metagenomic	SFB	240	19079	6678	1685	2428	FAO83526	LSK109
112.1	DNA	R9	MinIon	509	1842	34	metagenomic	SFB	474	24501	6387	2150	3460	FAO86805	LSK109
113.1	DNA	R9	MinIon	463	1637	87	metagenomic	SFB	189.6	3831	1777	2757	968	FAP46095	LSK109
114.1	DNA	R9	MinIon	488	1659	87	metagenomic	SFB	158.4	15254	5286	1071	1976	FAP49364	LSK109
115.1	DNA	R9	MinIon	500	1526	66	metagenomic	SFB	286.8	10070	3581	2934	1208	FAQ01716	LSK109
116.1	DNA	R9	MinIon	479	1728	52	metagenomic	SFB	470.4	6142	2766	2240	834	FAQ01897	LSK109
117.1	DNA	R9	MinIon	484	1680	52	metagenomic	SFB	477.6	7060	3049	1966	1301	FAQ10186	LSK109
118.1	DNA	R9	MinIon	495	1850	36	metagenomic	SFB	674.4	12282	5140	1137	1761	FAQ37458	LSK109
119.1	DNA	R9	MinIon	496	1817	37	metagenomic	SFB	574	11248	4596	1583	2062	FAQ69372	LSK109
120.1	DNA	R9	MinIon	499	1904	151	insect	LFB	103	2509	NA	13240	264	FAL78077	LSK109
120.2	DNA	R9	MinIon	481	1707	165	insect	SFB	113	5427	4328	5385	576	FAL78077	LSK109
121.1	DNA	R9	MinIon	509	1984	150	insect	LFB	232	5127	4677	10627	438	FAL83572	LSK109
122.1	DNA	R9	MinIon	380	1456	20	insect	SFB	255	2097	1977	5473	221	FAO10283	LSK109
10.1	DNA	R9	MinIon	508	NA	49	insect	LFB	144	2393	NA	20800	386	FAO10418	LSK109
10.3	DNA	R9	MinIon	390	1405	54	insect	LFB	144	1651	NA	21548	273	FAO10418	LSK109
10.4	DNA	R9	MinIon	354	1257	55	insect	SFB	255	1084	NA	5500	162	FAO10418	LSK109

ID (citation)	RNA/ DNA	fc version	device	#cha	#por	fc age	sample type	buffer	ng load	estimated bases (Mb)	EB12 (Mb)	mean rl	pore ht	fc id	library prep
123.1	DNA	R9	MinIon	462	1666	7	insect	SFB	226	7190	5361	4274	729	FAO10430	LSK109
123.2	DNA	R9	MinIon	373	1394	21	insect	SFB	255	1473	1441	10126	165	FAO10430	LSK109
124.1	DNA	R9	MinIon	497	1843	69	insect	LFB	200	2766	2638	16530	263	FAO12153	LSK109
124.2	DNA	R9	MinIon	447	1699	74	insect	LFB	200	2253	2205	16834	244	FAO12153	LSK109
124.3	DNA	R9	MinIon	388	1512	75	insect	LFB	160	1771	NA	15219	229	FAO12153	LSK109
124.4	DNA	R9	MinIon	358	1433	76	insect	LFB	160	1413	NA	16750	226	FAO12153	LSK109
124.5	DNA	R9	MinIon	355	1340	88	insect	LFB	150	1716	NA	19073	249	FAO12153	LSK109
124.6	DNA	R9	MinIon	230	951	89	insect	LFB	100	1003	NA	14562	258	FAO12153	LSK109
125.1	DNA	R9	MinIon	436	1540	60	insect	LFB	300	3879	3655	16927	361	FAO12159	LSK109
125.2	DNA	R9	MinIon	410	1489	61	insect	LFB	200	2721	2671	18638	272	FAO12159	LSK109
125.3	DNA	R9	MinIon	416	1591	61	insect	LFB	200	3097	NA	13451	547	FAO12159	LSK109
125.4	DNA	R9	MinIon	364	1366	62	insect	LFB	200	2271	NA	14192	338	FAO12159	LSK109
125.5	DNA	R9	MinIon	321	1196	67	insect	LFB	200	1319	NA	16557	175	FAO12159	LSK109
125.6	DNA	R9	MinIon	264	1086	68	insect	LFB	100	719	NA	16788	151	FAO12159	LSK109
126.1	DNA	R9	MinIon	510	1935	49	insect	SFB	200	9855	6485	4358	1016	FAO86712	LSK109
126.2	DNA	R9	MinIon	476	1811	50	insect	SFB	100	6855	5140	4640	724	FAO86712	LSK109
126.3	DNA	R9	MinIon	419	1570	51	insect	SFB	67	4758	4070	4859	545	FAO86712	LSK109
126.4	DNA	R9	MinIon	307	1381	62	insect	LFB	70	1701	1587	7019	264	FAO86712	LSK109
127.1	DNA	R9	MinIon	445	1709	8	insect	LFB	200	4467	4156	18700	443	FAO86834	LSK109
127.2	DNA	R9	MinIon	468	1785	9	insect	LFB	170	3610	3305	18665	336	FAO86834	LSK109
128.1	DNA	R9	MinIon	383	1424	6	insect	LFB	200	1543	1510	19752	113	FAO88446	LSK109
129.1	DNA	R9	MinIon	487	1796	55	insect	LFB	190	4379	3694	6359	357	FAO88464	LSK109
129.2	DNA	R9	MinIon	279	1046	57	insect	LFB	50	1507	NA	6788	311	FAO88464	LSK109
130.1	DNA	R9	MinIon	511	1944	44	insect	SFB	200	9475	6638	4590	922	FAO91712	LSK109
130.2	DNA	R9	MinIon	464	1753	48	insect	SFB	100	6429	4884	4961	692	FAO91712	LSK109
130.3	DNA	R9	MinIon	425	NA	51	insect	SFB	67	2592	2592	4828	525	FAO91712	LSK109
67.1	DNA	R9	MinIon	499	1907	8	insect	LFB	200	4891	4586	18872	455	FAO92001	LSK109
67.2	DNA	R9	MinIon	485	1852	9	insect	LFB	170	4284	3895	19414	417	FAO92001	LSK109
67.3	DNA	R9	MinIon	473	1776	13	insect	LFB	140	3997	3559	18674	368	FAO92001	LSK109
67.5	DNA	R9	MinIon	427	1508	44	insect	SFB	78	4948	3728	3529	607	FAO92001	LSK109
67.6	DNA	R9	MinIon	111	758	51	insect	LFB	30	648	578	14885	408	FAO92001	LSK109
131.1	DNA	R9	MinIon	512	1959	55	insect	LFB	190	7742	6090	6314	803	FAO92100	LSK109
131.2	DNA	R9	MinIon	498	1864	57	insect	LFB	100	3275	NA	6876	NA	FAO92100	LSK109
131.3	DNA	R9	MinIon	469	1772	57	insect	LFB	85	5929	4737	4932	894	FAO92100	LSK109
131.4	DNA	R9	MinIon	380	1421	58	insect	LFB	60	3028	2746	6700	370	FAO92100	LSK109
131.5	DNA	R9	MinIon	223	874	62	insect	LFB	70	807	778	6584	264	FAO92100	LSK109
132.1	DNA	R9	MinIon	465	1812	57	insect	LFB	100	4724	4089	7116	498	FAO92220	LSK109
132.2	DNA	R9	MinIon	359	1379	58	insect	LFB	60	2643	2390	6769	334	FAO92220	LSK109
133.1	DNA	R9	MinIon	506	1951	58	insect	LFB	190	6564	5533	6795	688	FAO92291	LSK109
133.2	DNA	R9	MinIon	433	1628	62	insect	LFB	85	3866	3453	7461	426	FAO92291	LSK109
134.1	DNA	R9	MinIon	488	1725	72	plant	SFB	350	8694	3799	2288	620	FAR96897	LSK109
135.1	DNA	R9	MinIon	473	1615	72	plant	SFB	330	9350	3929	4101	532	FAR96905	LSK110
136.1	RNA	R9	MinIon	511	1399	128	mouse	RNA	1252	1407	910	1058	915	FAO83512	RNA002
137.1	RNA	R9	MinIon	509	1383	128	mouse	RNA	1372	832	705	746	638	FAO92305	RNA002
138.1	RNA	R9	MinIon	494	1275	12	mouse	RNA	1092	1206	680	733	1094	FAP46096	RNA002
139.1	RNA	R9	MinIon	474	1263	12	mouse	RNA	1252	1174	708	772	1176	FAP46400	RNA002
140.1	RNA	R9	MinIon	501	1353	12	mouse	RNA	1220	1940	888	1049	1390	FAP46452	RNA002
141.1	RNA	R9	MinIon	486	1320	12	mouse	RNA	1044	1337	800	1091	827	FAP49780	RNA002
142.1	DNA	R9	MinIon	494	NA	86	mouse	SFB	385	10671	6305	10446	1935	FAQ58673	LSK109
142.2	DNA	R9	MinIon	NA	1236	NA	mouse	SFB	385	NA	2280	9714	370	FAQ58673	LSK109
143.1	DNA	R9	MinIon	479	1827	63	mouse	SFB	300	6680	4769	8821	599	FAQ58674	LSK109
144.1	DNA	R9	MinIon	447	1714	57	mouse	SFB	300	5561	4246	9428	612	FAQ61173	LSK109
145.1	DNA	R9	MinIon	499	NA	83	mouse	SFB	245	11137	6360	10034	1800	FAQ61176	LSK109
145.2	DNA	R9	MinIon	NA	1205	NA	mouse	SFB	245	NA	2779	9350	510	FAQ61176	LSK109
146.1	DNA	R9	MinIon	508	1949	63	mouse	SFB	282	21067	6967	9473	1993	FAQ69208	LSK109
147.1	DNA	R9	MinIon	508	1875	83	mouse	SFB	245	19740	6786	9155	1855	FAQ69274	LSK109
148.1	DNA	R9	MinIon	499	1909	83	mouse	SFB	385	19034	6723	9377	1832	FAQ69338	LSK109
149.1	DNA	R9	MinIon	507	1895	49	mouse	SFB	270	24681	7030	6962	2518	FAQ69370	LSK109
150.1	DNA	R9	MinIon	490	1829	57	mouse	SFB	280	22628	6920	8963	2237	FAQ69578	LSK109
151.1	DNA	R9	MinIon	509	1964	170	human	SFB	444	18354	6760	1550	2234	FAL84151	LSK109
151.2	DNA	R9	MinIon	213	728	172	human	SFB	444	2207	1506	1485	737	FAL84151	LSK109
152.1	RNA	R9	MinIon	505	1360	47	human	RNA	42	157	117	475	431	FAO10420	RNA002
153.1	DNA	R9	MinIon	502	NA	68	human	SFB	538	12217	6859	2414	1762	FAP46521	LSK109
154.1	DNA	R9	MinIon	489	NA	43	human	SFB	451	10568	6294	4487	1612	FAP49434	LSK109
154.2	DNA	R9	MinIon	448	1708	44	human	SFB	451	13249	5635	4181	1369	FAP49434	LSK109
155.1	DNA	R9	MinIon	NA	1919	NA	human	SFB	688.8	NA	6763	4626	2319	FAP49725	LSK109
156.1	DNA	R9	MinIon	501	1928	189	human	LFB	400	12833	NA	5179	1386	FAQ01752	LSK109
157.1	DNA	R9	MinIon	467	1430	57	human	SFB	382	7352	4147	515	712	FAQ09810	LSK109
158.1	DNA	R9	MinIon	480	1356	49	human	SFB	821	6529	4541	454	899	FAQ10598	LSK109
158.2	DNA	R9	MinIon	187	536	50	human	SFB	547	2326	1799	467	791	FAQ10598	LSK109
158.3	DNA	R9	MinIon	76	239	51	human	SFB	274	299	227	854	NA	FAQ10598	LSK109
159.1	DNA	R9	MinIon	488	1878	162	human	SFB	300	13259	5853	2776	1275	FAQ69371	LSK109
160.1	DNA	R9	MinIon	476	NA	92	human	SFB	400	15275	5655	2746	1314	FAR29925	LSK110
161.1	DNA	R9	MinIon	NA	1690	238	human	LFB	405	NA	4401	2257	544	FAR33296	LSK110
161.2	DNA	R9	MinIon	293	866	NA	human	LFB	405	2509	1905	927	350	FAR33296	LSK110
162.1	DNA	R9	MinIon	509	NA	123	human	LFB	394	19595	NA	NA	1491	FAR33573	LSK110
163.1	DNA	R9	MinIon	456	NA	130	human	LFB	305	8441	NA	643	759	FAR38117	LSK110
164.1	DNA	R9	MinIon	505	NA	27	human	LFB	365	9027	NA	NA	935	FAR39219	LSK109
165.1	DNA	R9	MinIon	498	NA	37	human	LFB	321	7285	NA	765	750	FAR39290	LSK109

ID (citation)	RNA/ DNA	fc version	device	#cha	#por	fc age	sample type	buffer	ng load	estimated bases (Mb)	EB12 (Mb)	mean rl	pore ht	fc id	library prep
166.1	DNA	R9	MinIon	508	NA	74	human	LFB	453	6073	NA	944	834	FAR39317	LSK110
167.1	DNA	R9	MinIon	377	1197	242	human	LFB	340	3463	2744	677	356	FAR39502	LSK110
168.1	DNA	R9	MinIon	490	NA	91	human	SFB	400	17476	5708	4673	1212	FAR93058	LSK110
169.1	DNA	R9	MinIon	383	993	247	human	LFB	340	2266	1726	848.	221	FAR96341	LSK110
170.1	DNA	R9	MinIon	481	1833	92	human	SFB	400	15910	5763	5055	1078	FAR96408	LSK110
171.1	DNA	R9	MinIon	483	1777	91	human	SFB	400	10105	4151	2782	1001	FAR96434	LSK110
172.1	DNA	R9	MinIon	384	1080	249	human	LFB	425	3106	2509	1029	397	FAR96514	LSK110
173.1	DNA	R9	MinIon	510	1882	37	human	SFB	300	7049	5165	1412	718	FAR96669	LSK109
173.2	DNA	R9	MinIon	300	1079	38	human	SFB	200	5071	2886	1414	726	FAR96669	LSK109
174.1	DNA	R9	MinIon	479	1294	238	human	LFB	389	5313	3079	849	446	FAR96893	LSK110
175.1	DNA	R9	MinIon	438	1308	171	human	LFB	405	3491	2671	947	630	FAS60674	LSK110
176.1	DNA	R9	MinIon	509	NA	90	human	LFB	450	20857	6391	587	2090	FAV38963	LSK110
176.2	DNA	R9	MinIon	NA	728	91	human	LFB	411	NA	1729	1164	584	FAV38963	LSK110
177.1	DNA	R9	MinIon	NA	NA	69	human	LFB	400	NA	NA	NA	NA	FAV38989	LSK110
177.2	DNA	R9	MinIon	452	1445	NA	human	LFB	330	8250	4531	907	984	FAV38989	LSK110
178.1	DNA	R9	MinIon	511	NA	60	human	LFB	400	14634	5579	622	1222	FAV39381	LSK110
179.1	DNA	R9	MinIon	NA	1437	NA	human	LFB	412	NA	6287	679	1644	FAV87040	LSK110
180.1	DNA	R9	MinIon	NA	1371	NA	human	LFB	414.96	NA	5864	569	1207	FAW80737	LSK110
9.2	RNA	R9	MinIon	270	694	83	synthetic	RNA	230	505	362	893	801	FAO07649	RNA002
124.7	RNA	R9	MinIon	189	484	103	synthetic	RNA	207	201	183	1002	469	FAO12153	LSK109
125.7	RNA	R9	MinIon	200	522	103	synthetic	RNA	212	195	179	1038	418	FAO12159	LSK109

Table S2. Overview of methylation calling tools (adapted from Liu *et al.*), sorted by publication date. 5mC – 5-methylcytosine in DNA; m5C – 5-methylcytosine in RNA; 6mA – N6-methyladenosine in DNA; m6A – N6-methyladenosine in RNA; 5hmC – 5-hydroxymethylcytosine; psU – pseudouridine; m1A – N1-methyladenosine; 5moU – 5-methoxyuridine; m7G – N7-methylguanosine; Ino – inosine; f5C – 5-formylcytidine; **mC – 2'-O-methylcytosine: mG – 2'-O-methylguanine; mA – 2'-O-methyladenosine; mU – 2'-O-methyluracil;**

comp. approach	tool	DNA / RNA	modification type	year	citation
hidden Markov models	`Nanopolish`	DNA	5mC	2017	(99)
statistical	`Tombo`	DNA / RNA	4mC, 5mC, m5C, 6mA	2017	(111)
statistical	`SignalAlign`	DNA	5mC, 5hmC, 6mA	2017	(112)
statistical	`NanoMod`	DNA	5mC	2019	(113)
neural networks	`mCaller`	DNA	6mA	2019	(114)
neural networks	`DeepSignal`	DNA	5mC, 6mA	2019	(115)
neural networks	`DeepMod`	DNA	5mC, 6mA	2019	(116)
neural network	`DeepMod2`	DNA	5mC	2024	(117)
hidden Markov models	`f5c`	DNA	5mC	2020	(95)
neural networks	`methBERT`	DNA	5mC, 6mA	2021	(118)
meta approaches	`METEORE`	DNA	5mC, 6mA	2021	(25)
neural networks	`DeepMP`	DNA	5mC, 6mA	2021	(119)
statistical	`-`	DNA	D2O	2024	(106)
neural networks	`Dorado`	DNA	4mC, 5mC, 5hmC, 6mA		ONT[1]
neural networks	`Megalodon`	DNA	5mC, 6mA		ONT[2]
machine learning	`MINES`	RNA	m6A	2019	(120)
error profile & machine learning	`EpiNano`	RNA	m6A, psU	2019	(121)
neural networks	`nanoDoc`	RNA	m6A	2020	(122)
neural networks	`nano-ID`	RNA	e5U	2020	(123)
statistical	`DRUMMER`	RNA	m6A	2020	(124)
statistical	`xPore`	RNA	m6A	2021	(125)
machine learning	`Nanom6A`	RNA	m6A	2021	(126)
statistical	`Yanocomp`	RNA	m6A	2021	(127)
machine learning	`nanoRMS`	RNA	psU	2021	(128)
error profile	`ELIGOS`	RNA	m6A, m1A, 5moU, psU, m7G, Ino, hm5C, f5C	2021	(129)
statistical	`Nanocompore`	RNA	m6A	2021	(130)
neural networks	`m6Anet`	RNA	m6A	2022	(131)
machine learning	`Penguin`	RNA	psU	2022	(132)
neural networks	`DENA`	RNA	m6A	2022	(133)
statistical	`Magnipore`	RNA	-	2023	(134)
neural networks	`mAFIA`	RNA	m6A	2024	(135)
neural networks	`Dorado`	RNA	mC, mG, mA, mU, m5C, m6A, Ino, psU		ONT[1]

[1] `https://github.com/nanoporetech/dorado`
[2] `https://github.com/nanoporetech/megalodon`

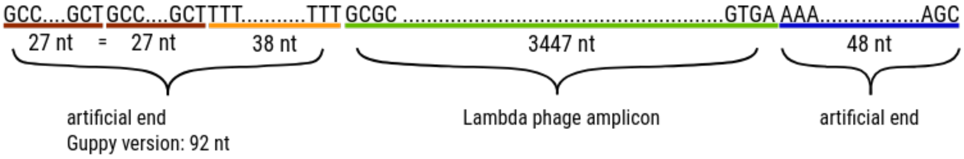

Figure S1. Structure of the ONT DNA spike-in: The positive control spike-in for DNA sequencing is a 3.6 kb amplicon, which maps to the 3’ end of the Lambda phage genome.

Table S3. Re-evaluated hand picked pore half time for different run IDs

ID	Half Life
1.1	998
2.1	1630
6.1	1510
10.1	386
88.1	3098
93.1	2041
110.1	1750
130.3	525
142.1	1935
145.1	1800
153.1	1762
154.1	1612
160.1	1314
162.1	1491
163.1	759
164.1	935
165.1	750
166.1	834
168.1	1212
178.1	1222
176.1	1971
176.1	2090

Table S4. AIC values for linear, polynomial and exponential models. We compared the model fit of linear model, second degree polynomial model and exponential model, and selected the model with the highest AIC. The models were tested for DNA and RNA seperately, comparing different variables against EB12. Based on the AIC values, we decided to use linear regression to analyze the influence of starting pores, starting channels and loaded library on EB12.

	AIC linear	AIC polynomial	AIC exponential
starting pores (DNA)	4 767.56	4 759.87	143.28
starting pores (RNA)	1 209.18	1 207.37	83.31
starting channels (DNA)	5 218.51	5 216.71	126.32
starting channels (RNA)	1 172.37	1 173.43	84.03
loaded library (DNA)	4 327.40	4 321.26	178.79
loaded library (RNA)	1 208.24	1 210.06	76.85

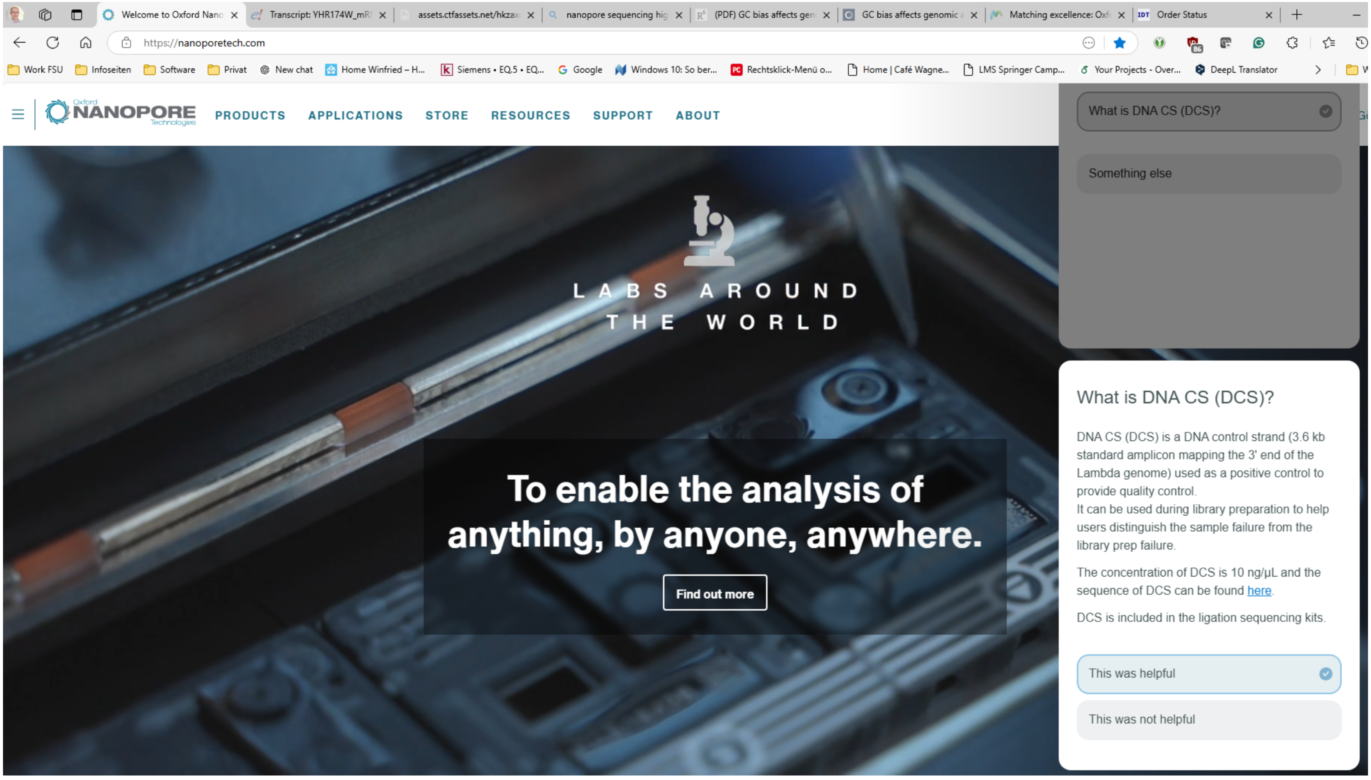

Figure S2. Screenshot from the Nanopore Community about DNA control sequence (DCS)

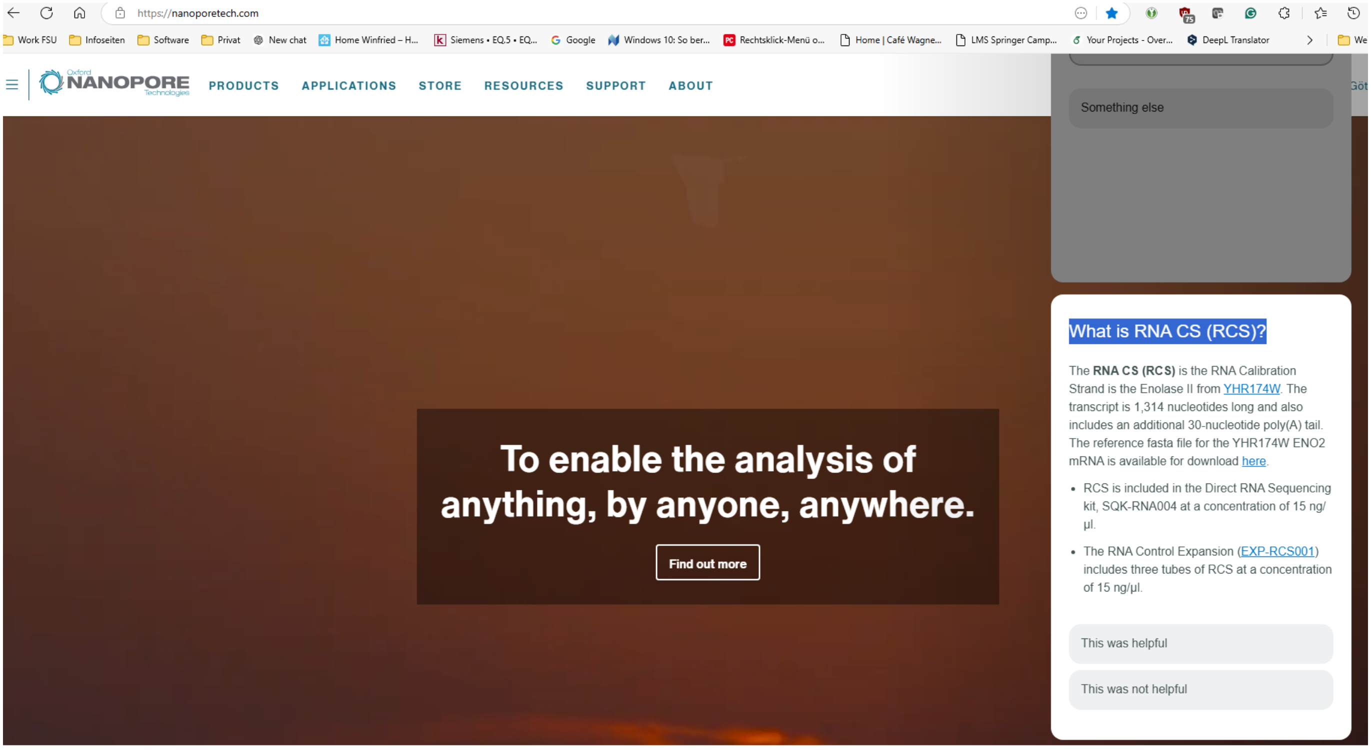

Figure S3. Screenshot from the Nanopore Community about RNA control sequence (RCS)

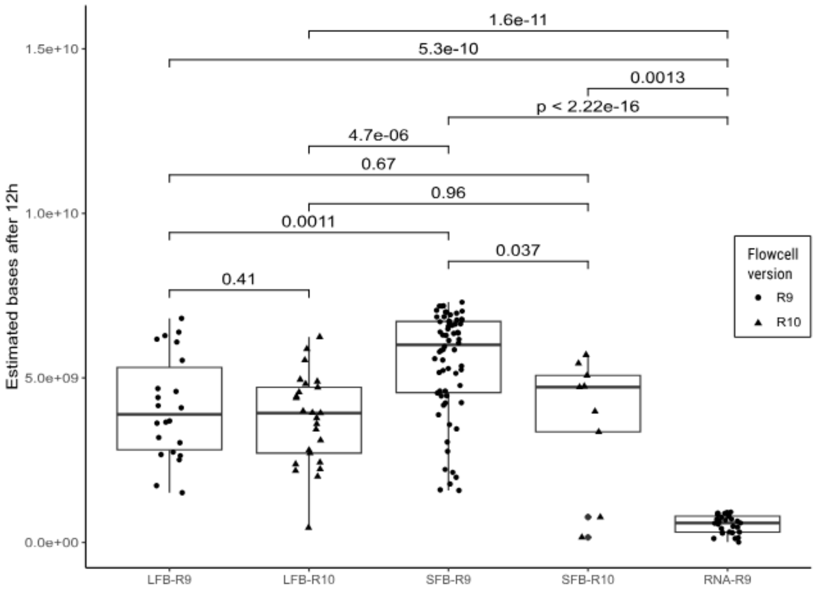

Figure S4. Seperate boxplots and t-tests for LFB-R9, LFB-R10, SFB-R9, SFB-R10 and RNA-R9 show significant differences between the used buffers for the R9 flowcells (LFB-R9 vs. SFB-R9 = 0.0011, LFB-R9 vs. RNA-R9 = $5.3 \cdot 10^{-10}$, SFB-R9 vs. RNA-R9 = $2.22 \cdot 10^{-16}$), but not for R10 (LFB-R10 vs. SFB-R10 = 0.96). A high variance for both SFB-R9 and LFB-R9 can be seen, which might result from different samples types as well as other influences discussed above. P-values were calculated using pairwise t-tests and are Bonferroni-adjusted to account for multiple comparisons.

Table S5. Testing the variables in a linear regression for their influence on EB12 without potential confounding variables changes their influence for some variables. No changes result for the variables starting pores, pore half time, and flow cell version, which have significant influence on EB12 both with and without potentially confounding variables. The influence of other variables is changed, revealing confounding effects. For the sample type, without integrating the potentially confounding variables, the virus sample type influences EB12, but when integrating all variables, the metagenomic sample type influences EB12. This effect arises from the fact that most virus samples are RNA samples. Also the influence of the sequencing buffer used seems to be confounded, as the RNA buffer strongly correlates with the sequencing material (RNA, DNA). Both median read length and starting channels occur only influential without the additional variables. Starting channel numbers depends on the amount of active pores in at the beginning of the sequencing.

	Estimate	Std. Error	t value	Pr(> |t|)	Significance
(Intercept)	-5.245e+09	6.743e+08	-7.777	1.81e-12	***
starting pores	5.713e+06	4.201e+05	13.599	< 2e-16	***
(Intercept)	1.492e+09	2.626e+08	5.683	7.24e-08	***
pore half time	1.863e+06	1.669e+05	11.161	< 2e-16	***
(Intercept)	4.742e+09	1.436e+08	33.03	< 2e-16	***
sequencing material - RNA	-4.188e+09	3.276e+08	-12.78	< 2e-16	***
(Intercept)	4.285e+09	3.625e+08	11.819	< 2e-16	***
sample type - human	3.211e+08	6.348e+08	0.506	0.614	
sample type - insect	7.920e+07	6.499e+08	0.122	0.903	
sample type - metagenomic	6.034e+08	4.804e+08	1.256	0.211	
sample type - mouse	-2.311e+08	6.348e+08	-0.364	0.716	
sample type - protist	-2.287e+08	1.072e+09	-0.213	0.831	
sample type - virus	-2.537e+09	5.127e+08	-4.948	2.07e-06	***
(Intercept)	3993942884	212058892	18.834	< 2e-16	***
flow cell version - R10	-248216052	446895007	-0.555	0.579	
(Intercept)	3.370e+09	2.480e+08	13.589	< 2e-16	***
median read length	2.064e+05	6.773e+04	3.048	0.00285	**
(Intercept)	-3.834e+09	1.460e+09	-2.626	0.0096	**
starting channels	1.622e+07	3.058e+06	5.305	4.23e-07	***
(Intercept)	3.906e+09	2.149e+08	18.178	< 2e-16	***
sequencing buffer – SFB	1.360e+09	2.741e+08	4.961	1.9e-06	***
sequencing buffer – RNA	-3.352e+09	3.479e+08	-9.635	< 2e-16	***
(Intercept)	3950771099	395305737	9.994	< 2e-16	***
library loaded [ng]	-73413	752851	-0.098	0.922	

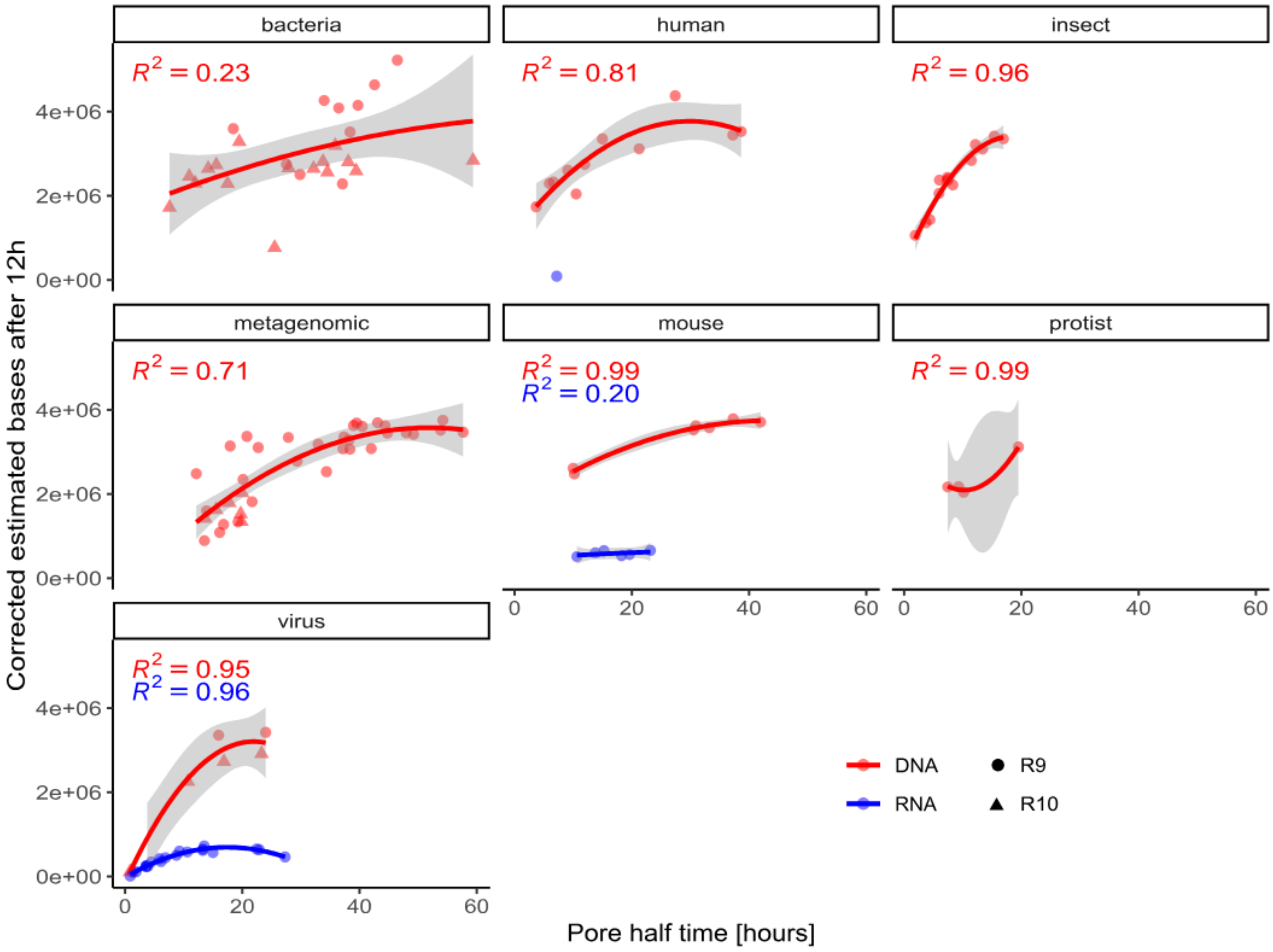

Figure S5. Correlation (R^2) between pore half time and the estimated number of bases after 12 hours of sequencing (EB12) is observed across most sample types. R^2 values are calculated for quadratic regression. The EB12 values were divided by number of starting pores, to corrected for any potential bias. The resulting plots reflect the same statements as Fig. 4A.

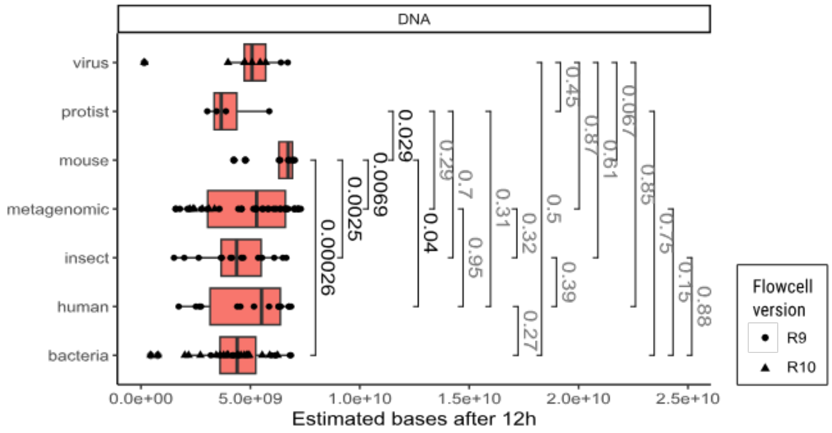

Figure S6. T-test shows higher EB12 in mouse compared to other sample types, addition to Fig. 4B. P-values were calculated using pairwise t-tests and are Bonferroni-adjusted to account for multiple comparisons. P-values above 0.05 are shown in grey.

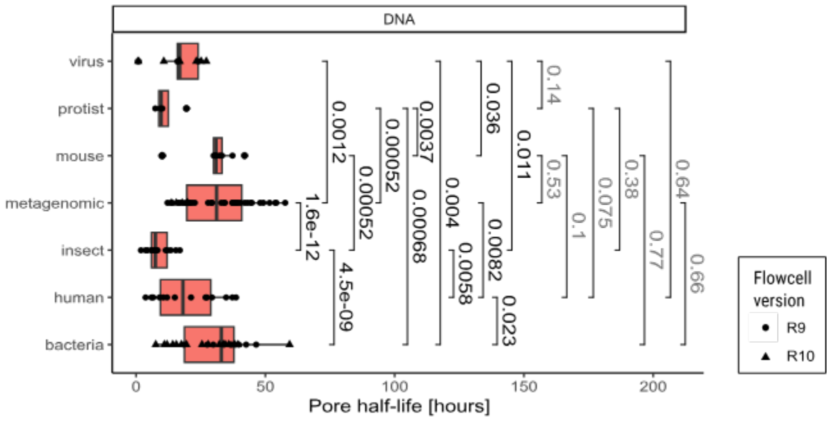

Figure S7. T-test shows significant differences in pore half time between sample types; addition to Fig. 4C. P-values were calculated using pairwise t-tests and are Bonferroni-adjusted to account for multiple comparisons. P-values above 0.05 are shown in grey.

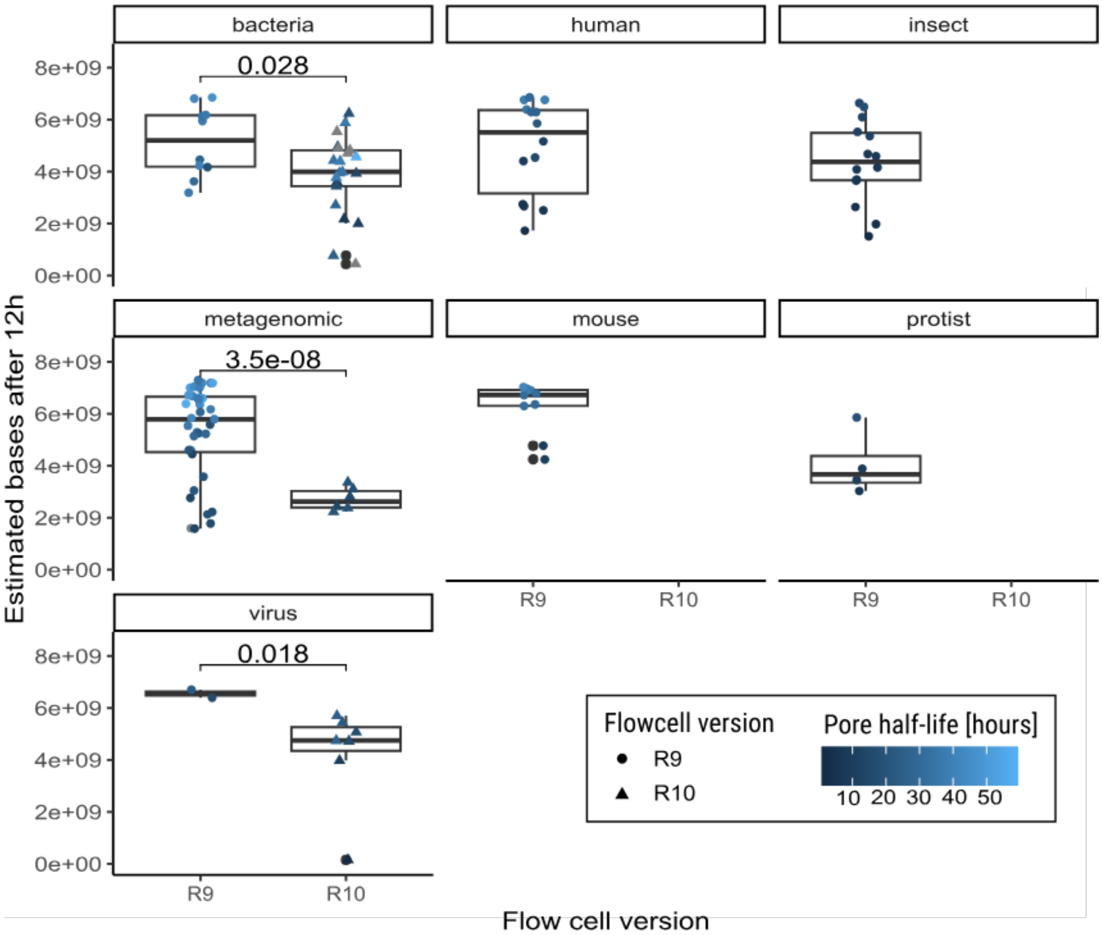

Figure S8. For the data shown in Fig. 4 B,C a t-test reveals significant differences in estimated bases after 12 hours between R9 and R10 data, when separating the data based on sample types. Additionally, to the reduced yield after 12 hours a reduced pore half time is visible for the R10 runs. Confounding factors could be different sample origins, the purity, and sequencing kit.

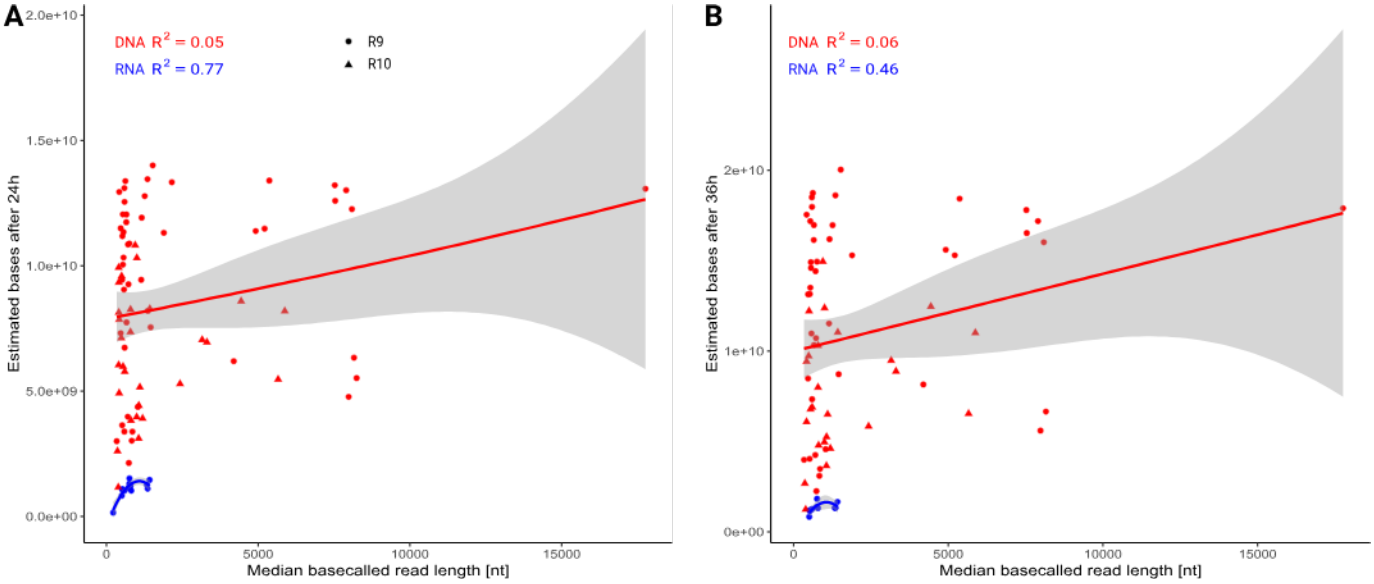

Figure S9. Distribution of estimated number of sequenced bases after 24 h (**A**) and 36 h (**B**) for DNA (red) and RNA (blue) samples; addition to Fig. 4D.

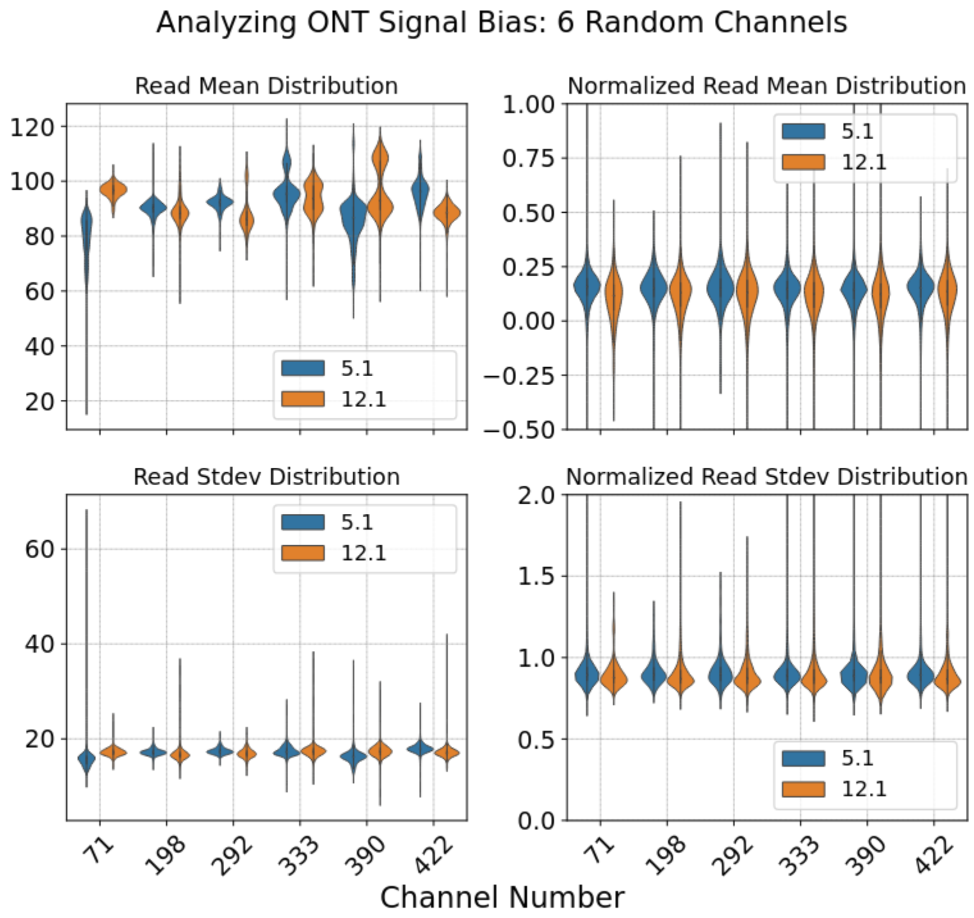

Figure S10. Read mean distribution (top) and read standard deviation (bottom) before (left) and after (right) normalization of six randomly picked channels from the sequencing runs 5.1 and 12.1. Biases caused by pores, flow cells, or sensors vanish after normalization.

ACCESS RAW ONT DATA

The following equation is used to convert the squiggles stored in the raw data to the pA signal:

$$\text{pA\_signal} = (\text{signal} + \text{offset}) * \text{range} / \text{digitization} \quad (1)$$

where `signal` represents the raw integer value, `offset` is the shift in the pA signal, `range` is the range of the pA signal, and `digitization` is the resolution of the conversion used to convert the continuous pA signal into discrete integer values. All parameters are provided by ONT in the raw data files.

NORMALIZATION

Normalization corrects for biases, ensuring that signals from different experiments or sequencing channels are directly comparable. It can be performed on each read individually using statistical measures such as the mean and standard deviation or the median and median absolute deviation (MAD). The median and MAD are more robust to outliers, which are common in sequencing data, see Fig. 8. ONT tools, including their basecallers, typically employ normalization during data processing. The standard formula for signal normalization, based on the median and MAD, is as follows:

$$\text{norm\_signal} = \frac{\text{pA\_signal} - \text{median}(\text{pA\_signal})}{\text{mad}(\text{pA\_signal})} \quad (2)$$

This process ensures that the signal distributions across reads become uniform, eliminating biases (see Fig. S10B).

Table S6. Sequencing statistics for the samples used in Fig. 5. cum. yield (Gb) – cumulative amount of estimated bases for all sequencing runs on this flow cell.

	ID 156	ID 124
#pores	1 928	1 843
#channels	501	497
# wash steps	2	6
cum. yield (Gb)	12.83	11.13
sample type	human	insect
fc version	R9	R9
device	MinION	MinION
RNA/ DNA	DNA	DNA

Table S7. Samples used for adaptive sampling in Fig. 6. # por – amount of active pores at sequencing start; yield – cumulative sequencing yield in Gb; depth – mean sequencing depth on CpG islands; wash – number of times the flow cell was washed in between; buffer – extension length of the CpG islands

id	# por	yield	depth	wash	buffer
NA	NA	10.7	3.01	0	0
NA	1519	19.7	6.10	0	0
155	1504	16.7	4.75	0	0
154	1305	23.3	6.67	1	0
153	1379	11.4	3.28	0	1
153	822	2.58	1.39	0	1
NA	1001	2.59	0.67	0	500
158	1192	8.65	2.13	2	500
157	1269	8.72	4.76	1	500
156	1250	13.49	3.85	2	0
164	1405	9.68	8.79	1	2000
165	1433	7.74	8.88	2	2000
166	1548	6.39	7.60	2	2000
162	1620	21.26	17.79	1	2000
163	969	9.00	7.24	1	2000
174	1352	6.46	5.40	1	2000
167	693	3.77	3.42	0	2000
169	749	2.60	2.13	0	2000
172	787	3.51	3.08	0	2000
161	1063	5.45	1.52	1	0
